# Supplementary material for: Test-trace-isolate-quarantine (TTIQ) intervention strategies after symptomatic COVID-19 case identification
Source: PLoS One. 2022 Feb 11;17(2):e0263597. doi: 10.1371/journal.pone.0263597 (PMC8836351; doi:10.1371/journal.pone.0263597)
Supplement: S1 Appendix — This file contains mathematical details of the model and analysis. (PDF) [file pone.0263597.s001.pdf]

S1 Appendix. Supplementary materials and methods

For manuscript "Test-trace-isolate-quarantine (TTIQ) intervention strategies after symptomatic COVID-19 case identification"

Peter Ashcroft[1*], Sonja Lehtinen[1], Sebastian Bonhoeffer[1*]

1 Institute of Integrative Biology, ETH Zurich, Switzerland

* peter.ashcroft@env.ethz.ch (PA); seb@env.ethz.ch (SB)

Generation times, infectivity profiles, and incubation periods

In our branching process model, the time at which an infector transmits SARS-CoV-2 to an infectee is determined from empirically-observed distributions. Concretely, the time at which an identified index case developed symptoms, t_{S_1}, is known, but the time at which they were infected, t_1, is generally unknown. Secondary contacts will be infected by the index case at some time t_2 ($t_2 > t_1$), and, if symptomatic, will develop symptoms at time t_{S_2}. These timepoints are illustrated in Fig IA.

The relationships between the times t_1, t_{S_1}, t_2, t_{S_2} are determined by: the generation time distribution, $q(t_2 - t_1|\theta_q)$, describing the time interval between the infection of an index case and secondary contact (Fig IB); the infectivity profile, $p(t_2 - t_{S_1}|\theta_p)$, describing the time interval between the onset of symptoms in the index case and infection of the secondary contact (Fig IC); and the incubation period distribution, $h(t_{S_1} - t_1)$, describing the time between the infection of an individual and the onset of their symptoms (Fig ID). For these distributions, we use empirical estimates from Ferretti et al. [1]. The parameters that define the generation time distribution, infectivity profile, and the incubation period distribution are shown in Table I.

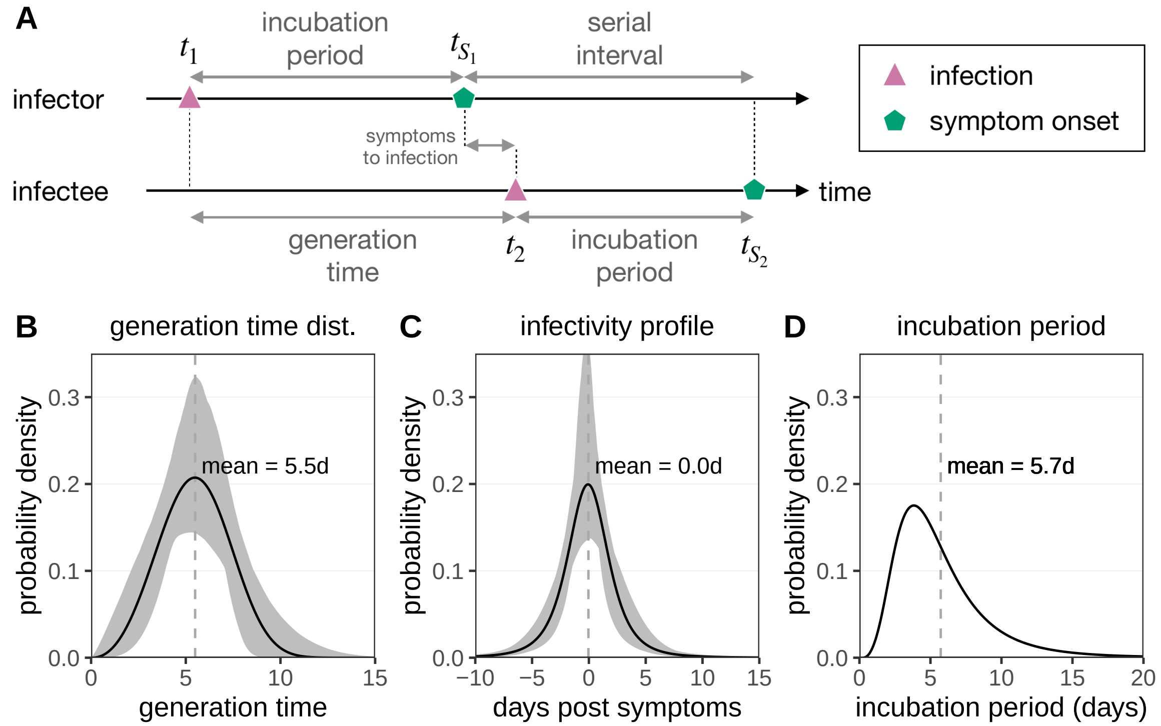

Fig I. Empirical distributions for infection time and symptom onset. A: The timeline of infection for an infector–infectee transmission pair. The infector (index case) is initially infected at time t_1, and after a period of incubation develops symptoms at time t_{S_1}. The infectee (secondary contact) is infected by the infector at time t_2, which can be before (presymptomatic infections) or after (symptomatic infection) t_{S_1}. The infectee then develops symptoms at time t_{S_2}. The generation time is then defined as $t_2 - t_1$ (the time between infections), while the serial interval is defined as $t_{S_2} - t_{S_1}$ (the time between symptom onsets). B: The generation time distribution $[q(t|\theta_q) = q(t_2 - t_1|\theta_q)]$ follows a Weibull distribution, and is inferred from the serial interval distribution [1]. C: The infectivity profile $[p(t|\theta_p) = p(t_2 - t_{S_1}|\theta_p)]$ follows a shifted Student's t-distribution, and is also inferred from the serial interval distribution [1]. D: The distribution of incubation times $[h(t) = h(t_{S_1} - t_1)]$ follows a meta-distribution constructed from the average of seven reported log-normal distributions, as described in Ferretti et al. [1] [2–8]. Data provided in S1 Dataset.

Table I. Parameters of the distributions used in this work to describe the timing of infection events.

Distribution	Shape	Properties	Parameters
Incubation period $h(t)$	Meta-log-normal	mean = 5.723, sd = 3.450, median = 4.936	meanlog = 1.570, sdlog = 0.650 (Bi [2])
			meanlog = 1.621, sdlog = 0.418 (Lauer [4])
			meanlog = 1.434, sdlog = 0.661 (Li [5])
			meanlog = 1.611, sdlog = 0.472 (Linton [6])
			meanlog = 1.857, sdlog = 0.547 (Ma [7])
			meanlog = 1.540, sdlog = 0.470 (Zhang [8])
			meanlog = 1.530, sdlog = 0.464 (Jiang [3])
Generation time $q(t\|\theta_q)$	Weibull	mean = 5.494, sd = 1.845, median = 5.479	shape = 3.277, scale = 6.127
Infectivity profile $p(t\|\theta_p)$	Shifted Student's t	mean = -0.042, sd = 2.876, median = -0.078	shift = -0.078, scale = 1.86, df = 3.35

The meta-log-normal incubation period distribution is the average of seven reported log-normal incubation period distributions as described by Ferretti et al. [1] [2–8]. The properties listed for the incubation period distribution are the mean, standard deviation (sd), and median of this meta-log-normal distribution. The shifted Student's t distribution for the infectivity profile is defined in `R` by `dt((x-shift)/scale, df)/scale` [1].

Asymptomatic versus symptomatic infections

We assume that a fraction a of all infections are persistently asymptomatic, with the remainder being classed as symptomatic (which includes individuals that are pre-symptomatic and post-symptom onset). Whether a new infectee is persistently-asymptomatic or not is assumed to be independent of whether the infector was persistently-asymptomatic or not. A meta-analysis has estimated a fraction $a \approx 20\%$ of infections are asymptomatic [9].

We now introduce parameters that describe the infectiousness of asymptomatic or symptomatic individuals. An asymptomatic individual would infect an average of R_a secondary contacts during their whole uninterrupted infectious period (i.e. in the absence of any TTIQ intervention, but in the presence of non-modelled interventions such as social distancing and hygiene protocols). A symptomatic individual will infect an average of R_s secondary contacts during their whole uninterrupted infectious period (i.e. no TTIQ). In general we have $R_a \neq R_s$, and we expect that $R_a \leq R_s$ based on empirical observations [9].

We can define the average reproductive number in the absence of TTIQ as

$$R = aR_a + (1-a)R_s, \tag{S1.1}$$

i.e. the average number of secondary infections per infected throughout the infectious period. The fraction of transmission that is attributable to asymptomatic individuals in the absence of TTIQ is then defined as

$$\alpha = \frac{aR_a}{aR_a + (1-a)R_s} = \frac{aR_a}{R}. \tag{S1.2}$$

Note that for $R_a = R_s$ (equal transmission from asymptomatics and symptomatics), we have $\alpha = a$. For $R_a < R_s$, we have $\alpha < a$. As α must be a positive number, we can bound the fraction of transmission from asymptomatic individuals in the absence of TTIQ by the limits $0 \leq \alpha \leq a$.

Although we modify the relative infectiousness of asymptomatic versus symptomatic individuals, we assume that the distribution of infection times is equal for both classes.

Quantifying secondary infections under TTIQ

Consider an infected individual who develops symptoms of COVID-19 at time t_{S_1}. The time at which this individual was infected, $t_1 < t_{S_1}$, is generally unknown. Without any TTIQ intervention this symptomatic individual would contact and infect R_s individuals during the course of the infection. The number of secondary infections up to a time T_1 after developing symptoms would then be

$$R_s \int_{-\infty}^{T_1} \mathrm{d}t_2 \, p(t_2 - t_{S_1}|\theta_p) = R_s P(T_1 - t_{S_1}|\theta_p), \tag{S1.3}$$

where $p(t|\theta_p)$ is the infectivity profile and $P(t|\theta_p) = \int_{-\infty}^{t} \mathrm{d}t' \, p(t'|\theta_p)$ is the cumulative infectivity profile.

Infected individuals who develop symptoms and/or test positive for SARS-CoV-2 should be

isolated from the population. In our model this occurs in a fraction f of symptomatic individuals who are then isolated at a time $T_1 = t_{S_1} + \Delta_1$, where $\Delta_1 > 0$ is the delay between symptom onset and isolation. The parameter Δ_1 can be interpreted as the delay of taking a test after symptom onset, waiting for the result, and entering isolation, or alternatively as the delay between symptom onset and self-isolation. The remaining fraction $1 - f$ of symptomatic individuals, along with the asymptomatic individuals, are not isolated ($T_1 \to \infty$). We can compute the expected number of secondary infections, n_2, as a function of the asymptomatic fraction a, isolation probability f, and delay Δ_1, as shown in Fig II. We then have

$$n_2(f, \Delta_1|\theta_p) = aR_a + (1-a)\left[fP(\Delta_1|\theta_p)R_s + (1-f)R_s\right], \tag{S1.4}$$

where the first term represents the secondary infections caused by asymptomatic individuals (who cannot be isolated), the first term in the bracket represents the secondary infections caused by symptomatic index cases prior to their isolation, and the final term is the secondary infections caused by symptomatic individuals who are not isolated. Now replacing $aR_a = \alpha R$ and $(1-a)R_s = (1-\alpha)R$ [from Eq (S1.2)], we can rearrange Eq (S1.4) to give

$$n_2(f, \Delta_1|\theta_p) = R\left[(1-\alpha)fP(\Delta_1|\theta_p) + (1-(1-\alpha)f)\right]. \tag{S1.5}$$

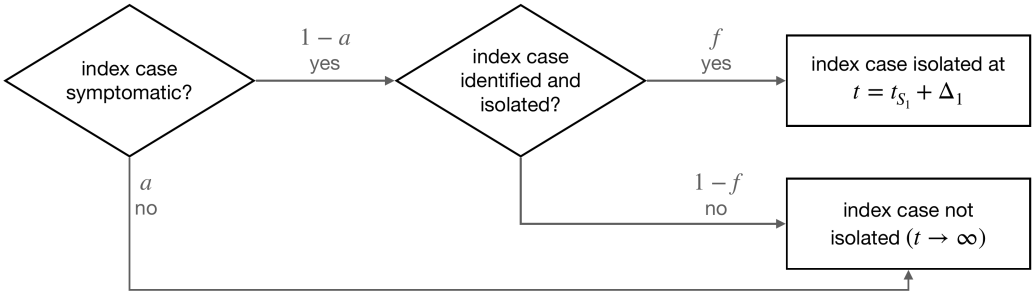

Fig II. Flowchart for computing the number of secondary infections under testing & isolation.

Quantifying tertiary infections under TTIQ

Each infected secondary contact has the potential to cause further infections, which will be the tertiary contacts of the initial infected. The number of infections caused by a secondary contact who is infected at t_2 and isolated at time T_2, will be

$$R_\bullet \int_{t_2}^{T_2} \mathrm{d}t_3\, q(t_3 - t_2|\theta_q) = R_\bullet Q(T_2 - t_2|\theta_q), \tag{S1.6}$$

where $R_\bullet \in \{R_a, R_s\}$ is the number of infections per secondary contact during the uninterrupted infectious period, t_3 is the infection time of the tertiary contacts, $q(t|\theta_q)$ is the generation time distribution, and $Q(t|\theta_q) = \int_0^t \mathrm{d}t'\, q(t'|\theta_q)$ is the cumulative generation time distribution. Note that we use the generation time distribution here, as our reference point is the time of infection (t_2),

whereas in Eq (S1.5) the reference point was the time of symptom onset (t_{S_1}).

Under TTIQ interventions, the symptomatic index and secondary cases can be isolated following a positive test result after symptom onset. If an index case is confirmed positive, then contact tracing can be used to identify and quarantine individuals who have recently been exposed to the confirmed case. Quarantining these individuals prevents the onward infection of tertiary contacts (Fig 1B in the manuscript). Importantly, whether an individual is quarantined is independent of symptom status. We introduce three further parameters to quantify contact tracing and quarantine: i) $\tau > 0$, the duration of lookback prior to symptom onset of the index case in which contacts are traced; ii) $0 \leq g \leq 1$, the probability to identify and quarantine a secondary contact that was infected within the contact tracing window; and iii) $\Delta_2 > 0$, the delay between isolating the index case and quarantining the identified secondary contacts.

There are many permutations of events that contribute to the number of tertiary infections under TTIQ, as shown in Fig III. The index case may not be detected due to being asymptomatic (a), or being symptomatic but not tested ($(1-a)(1-f)$), and hence contact tracing is not possible. If the index case is symptomatic and detected ($(1-a)f$), then a fraction g of the secondary contacts that were infected within the contact tracing window ($t_{S_1} - \tau \leq t_2 \leq t_{S_1} + \Delta_1$) are quarantined at time $t_{S_1} + \Delta_1 + \Delta_2$ (as shown in Fig 1B in the manuscript). The remaining fraction $1-g$ of secondary contacts, as well as the secondary contacts that were infected outside of the contact tracing window ($t_2 < t_{S_1} - \tau$), are not quarantined. However, the non-traced contacts may themselves become symptomatic and, after testing, become index cases that are isolated at time $t_{S_2} + \Delta_1$, where t_{S_2} is the symptom onset time of the secondary case. By considering these different scenarios, we arrive at an expression for the number of tertiary infections per index case under TTIQ,

$$\begin{aligned}
&n_3(f, \Delta_1, \tau, g, \Delta_2 | t_{S_1}, t_{S_2}, \theta_p, \theta_q) = \\
&\quad R_s(1-a)fg \int_{t_{S_1}-\tau}^{t_{S_1}+\Delta_1} \mathrm{d}t_2\, p(t_2 - t_{S_1}|\theta_p) RQ(t_{S_1} + \Delta_1 + \Delta_2 - t_2|\theta_q) + \\
&\quad R_s(1-a)f(1-g) \int_{t_{S_1}-\tau}^{t_{S_1}+\Delta_1} \mathrm{d}t_2\, p(t_2 - t_{S_1}|\theta_p) \psi(f, t_{S_2} + \Delta_1 - t_2|\theta_q) + \\
&\quad R_s(1-a)f \int_{-\infty}^{t_{S_1}-\tau} \mathrm{d}t_2\, p(t_2 - t_{S_1}|\theta_p) \psi(f, t_{S_2} + \Delta_1 - t_2|\theta_q) + \\
&\quad [aR_a + (1-a)R_s(1-f)] \int_{-\infty}^{\infty} \mathrm{d}t_2\, p(t_2 - t_{S_1}|\theta_p) \psi(f, t_{S_2} + \Delta_1 - t_2|\theta_q),
\end{aligned} \tag{S1.7}$$

where the shorthand

$$\psi(f, t_{S_2} + \Delta_1 - t_2|\theta_q) = \left[aR_a + (1-a)R_s\big(fQ(t_{S_2} + \Delta_1 - t_2|\theta_q) + (1-f)\big)\right] \tag{S1.8}$$

is the expected number of onward infections caused by each non-quarantined secondary contact. Each row in Eq (S1.7) corresponds to: i) tertiary infections caused by secondary contacts prior to their quarantine; ii) tertiary infections caused by secondary contacts who could have been quarantined but were not; iii) tertiary infections caused by secondary contacts who were infected before the quarantine window, and hence are not quarantined; iv) tertiary infections caused by secondary contacts who were infected by non-identified index cases.

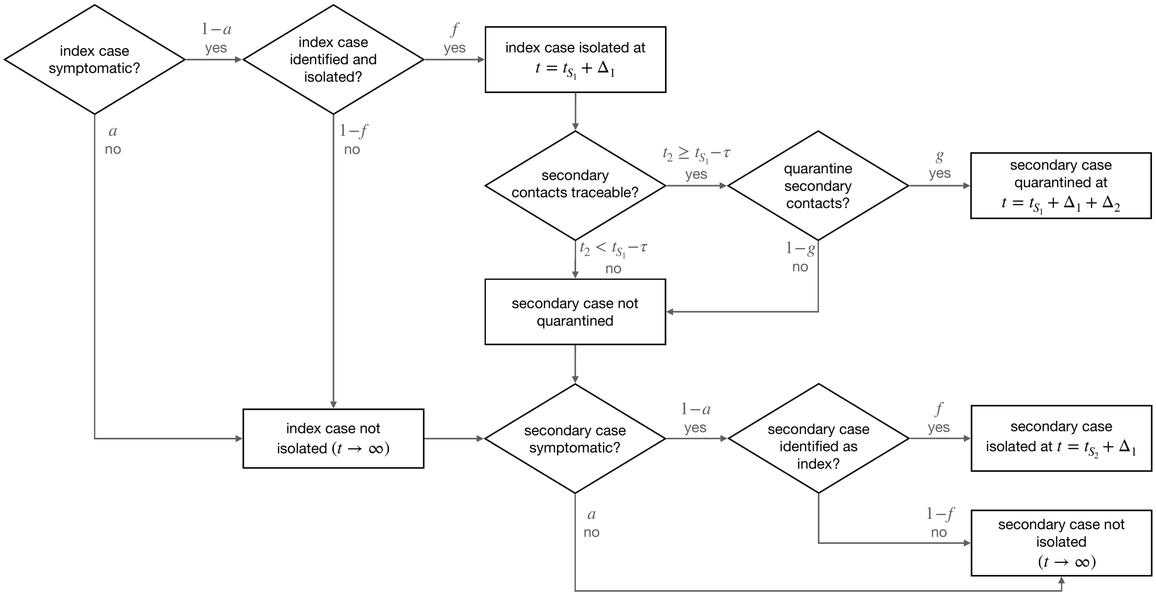

Fig III. Flowchart for computing the number of tertiary infections under TTIQ.

We now have to average Eq (S1.7) over t_{S_2} to obtain the expected number of tertiary infections per index case under TTIQ. We first note that $t_{S_2} = t_2 + \gamma$ for incubation period $\gamma \geq 0$. Hence we can write

$$\left\langle Q(t_{S_2} + \Delta_1 - t_2|\theta_q)\right\rangle_{t_{S_2}} = \int_0^\infty \mathrm{d}\gamma\, h(\gamma) Q(\gamma + \Delta_1|\theta_q), \tag{S1.9}$$

where $h(\gamma)$ is the incubation period distribution. We define the quantity

$$J(\Delta_1|\theta_q) = \left\langle Q(t_{S_2} + \Delta_1 - t_2|\theta_q)\right\rangle_{t_{S_2}}. \tag{S1.10}$$

Note that we have assumed the independence between symptom onset and infectivity, which may lead to an overestimation of the fraction of tertiary infections prevented.

Keeping t_{S_1} fixed as the reference time point, averaging Eq (S1.7) over t_{S_2} gives the expected number of tertiary infections per infected under TTIQ:

$$\begin{aligned}
&n_3(f, \Delta_1, \tau, g, \Delta_2|\theta_p, \theta_q) = \\
&\quad R_s(1-a)fgR\int_{-\tau}^{\Delta_1} \mathrm{d}t'\, p(t'|\theta_p) Q(\Delta_1 + \Delta_2 - t'|\theta_q) + \\
&\quad R_s(1-a)f(1-g)\big[P(\Delta_1|\theta_p) - P(-\tau|\theta_p)\big]\left[aR_a + (1-a)R_s\big(fJ(\Delta_1|\theta_q) + (1-f)\big)\right] + \\
&\quad R_s(1-a)fP(-\tau|\theta_p)\left[aR_a + (1-a)R_s\big(fJ(\Delta_1|\theta_q) + (1-f)\big)\right] + \\
&\quad \left[aR_a + (1-a)R_s(1-f)\right]\left[aR_a + (1-a)R_s\big(fJ(\Delta_1|\theta_q) + (1-f)\big)\right],
\end{aligned} \tag{S1.11}$$

where we have substituted $t' = t_2 - t_{S_1}$ such that

$$\int_{t_{S_1}-\tau}^{t_{S_1}+\Delta_1} \mathrm{d}t_2\, p(t_2 - t_{S_1}|\theta_p) Q(t_{S_1} + \Delta_1 + \Delta_2 - t_2|\theta_q) = \int_{-\tau}^{\Delta_1} \mathrm{d}t'\, p(t'|\theta_p) Q(\Delta_1 + \Delta_2 - t'|\theta_q). \tag{S1.12}$$

Now replacing $aR_a = \alpha R$ and $(1-a)R_s = (1-\alpha)R$ [from Eq (S1.2)], Eq (S1.11) can be further simplified to

$$\begin{aligned} &n_3(f, \Delta_1, \tau, g, \Delta_2|\theta_p, \theta_q) = \\ &\quad R^2(1-\alpha)fg \int_{-\tau}^{\Delta_1} \mathrm{d}t'\, p(t'|\theta_p) Q(\Delta_1 + \Delta_2 - t'|\theta_q) + \\ &\quad R^2\big[(1-\alpha)f(1-g)P(\Delta_1|\theta_p) + (1-\alpha)fgP(-\tau|\theta_p) + (1-(1-\alpha)f)\big] \times \\ &\qquad \big[(1-\alpha)fJ(\Delta_1|\theta_q) + (1-(1-\alpha)f)\big]. \end{aligned} \tag{S1.13}$$

Finally, in the absence of contact tracing ($g = 0$) Eq (S1.13) can be simplified, such that the number of tertiary infections per infected under testing & isolation only is given by

$$\begin{aligned} n_3(f, \Delta_1|\theta_p, \theta_q) = R^2\big[(1-\alpha)fP(\Delta_1|\theta_p) + (1-(1-\alpha)f)\big] \times \\ \big[(1-\alpha)fJ(\Delta_1|\theta_q) + (1-(1-\alpha)f)\big]. \end{aligned} \tag{S1.14}$$

From Eqs (S1.13) and (S1.14), we observe that the parameter f is always coupled to $1-\alpha$. We could therefore define a new parameter $\phi = (1-\alpha)f$ as the fraction of all infecteds that are isolated (as opposed to f which is the fraction of symptomatic infecteds isolated) to simplify our expressions. However, we choose to keep α and f explicitly in the calculations for clarity.

As a final point, we could repeat the derivation of Eq (S1.13), but this time only consider the number of tertiary infections that were caused by an asymptomatic secondary contact. I.e. we can calculate how much transmission is attributable to asymptomatics versus symptomatics in the presence of TTIQ. This leads to the expression

$$\begin{aligned} &n_3^{(\text{asymp})}(f, \Delta_1, \tau, g, \Delta_2|\theta_p, \theta_q) = \\ &\quad \alpha R^2(1-\alpha)fg \int_{-\tau}^{\Delta_1} \mathrm{d}t'\, p(t'|\theta_p) Q(\Delta_1 + \Delta_2 - t'|\theta_q) + \\ &\quad \alpha R^2\big[(1-\alpha)f(1-g)P(\Delta_1|\theta_p) + (1-\alpha)fgP(-\tau|\theta_p) + (1-(1-\alpha)f)\big]. \end{aligned} \tag{S1.15}$$

Reproductive number under TTIQ

For our branching process model, we define the reproductive number as

$$R_{\text{TTIQ}} = \frac{n_3(f, \Delta_1, \tau, g, \Delta_2|\theta_p, \theta_q)}{n_2(f, \Delta_1|\theta_p)}, \tag{S1.16}$$

where n_2 [Eq (S1.5)] and n_3 [Eq (S1.13)] are the expected number of secondary and tertiary infections per infected, respectively. In other words, we define the reproductive number as the

average number of infecteds in the third generation per infected in the second generation. It is necessary to work with the third generation (as opposed to just the first and second generations) as this is where the impact of contact tracing and quarantine is first observed.

Likewise, in the presence of testing & isolation only (i.e. no contact tracing & quarantine), the reproductive number is given by

$$R_{\mathrm{TI}} = \frac{n_3(f, \Delta_1|\theta_p, \theta_q)}{n_2(f, \Delta_1|\theta_p)}, \tag{S1.17}$$

where n_3 is now given by Eq (S1.14).

Confidence intervals

The primary sources of uncertainty in the outcomes of this model come from the generation time distribution and infectivity profile, which are inferred from empirical serial interval distributions [1]. Following Ferretti et al. [1], we use a likelihood ratio test to extract sample parameter sets for each distribution that lie within the 95% confidence interval.

Concretely, we first identify the maximum likelihood parameter sets $\hat{\theta}_p$ and $\hat{\theta}_q$ for the infectivity profile and generation time distribution, respectively. We then randomly sample the parameter space of each distribution, and keep 1,000 parameter sets whose likelihood satisfies $\ln \mathcal{L}(\theta) > \ln \mathcal{L}(\hat{\theta}) - \lambda_n/2$, where λ_n is the 95% quantile of a χ^2 distribution with n degrees of freedom. The infectivity profile is described a shifted Student's t-distribution, which has $n = 3$ parameters, while the generation time is described by a Weibull distribution with $n = 2$ parameters.

We then use these sampled parameter sets to generate R_{TTIQ}, and the extrema across all of these parameter sets determines the 95% confidence interval for the reproductive number under TTIQ. We need to use and combine estimates of both θ_p and θ_q. We assume parameter independence, and keep all (θ_p, θ_q) combinations whose joint likelihood satisfies $\ln \mathcal{L}(\theta_p) + \ln \mathcal{L}(\theta_q) > \ln \mathcal{L}(\hat{\theta}_p) + \ln \mathcal{L}(\hat{\theta}_q) - \lambda_5/2$.

References

1. Ferretti L, Ledda A, Wymant C, Zhao L, Ledda V, Dorner LA, et al. The Timing of COVID-19 Transmission. medRxiv. 2020; p. 2020.09.04.20188516. doi:10.1101/2020.09.04.20188516.

2. Bi Q, Wu Y, Mei S, Ye C, Zou X, Zhang Z, et al. Epidemiology and Transmission of COVID-19 in 391 Cases and 1286 of Their Close Contacts in Shenzhen, China: A Retrospective Cohort Study. The Lancet Infectious Diseases. 2020;20(8):911–919. doi:10.1016/S1473-3099(20)30287-5.

3. Jiang X, Niu Y, Li X, Li L, Cai W, Chen Y, et al. Is a 14-Day Quarantine Period Optimal for Effectively Controlling Coronavirus Disease 2019 (COVID-19)? medRxiv. 2020; p. 2020.03.15.20036533. doi:10.1101/2020.03.15.20036533.

4. Lauer SA, Grantz KH, Bi Q, Jones FK, Zheng Q, Meredith HR, et al. The Incubation Period of Coronavirus Disease 2019 (COVID-19) From Publicly Reported Confirmed Cases: Estimation and Application. Annals of Internal Medicine. 2020;172(9):577–582. doi:10.7326/M20-0504.

5. Li Q, Guan X, Wu P, Wang X, Zhou L, Tong Y, et al. Early Transmission Dynamics in Wuhan, China, of Novel Coronavirus–Infected Pneumonia. New England Journal of Medicine. 2020;382:1199–1207. doi:10.1056/NEJMoa2001316.

6. Linton NM, Kobayashi T, Yang Y, Hayashi K, Akhmetzhanov AR, Jung Sm, et al. Incubation Period and Other Epidemiological Characteristics of 2019 Novel Coronavirus Infections with Right Truncation: A Statistical Analysis of Publicly Available Case Data. Journal of Clinical Medicine. 2020;9(2):538. doi:10.3390/jcm9020538.

7. Ma S, Zhang J, Zeng M, Yun Q, Guo W, Zheng Y, et al. Epidemiological Parameters of Coronavirus Disease 2019: A Pooled Analysis of Publicly Reported Individual Data of 1155 Cases from Seven Countries. medRxiv. 2020; p. 2020.03.21.20040329. doi:10.1101/2020.03.21.20040329.

8. Zhang J, Litvinova M, Wang W, Wang Y, Deng X, Chen X, et al. Evolving Epidemiology and Transmission Dynamics of Coronavirus Disease 2019 Outside Hubei Province, China: A Descriptive and Modelling Study. The Lancet Infectious Diseases. 2020;20(7):793–802. doi:10.1016/S1473-3099(20)30230-9.

9. Buitrago-Garcia D, Egli-Gany D, Counotte MJ, Hossmann S, Imeri H, Ipekci AM, et al. Occurrence and Transmission Potential of Asymptomatic and Presymptomatic SARS-CoV-2 Infections: A Living Systematic Review and Meta-Analysis. PLOS Medicine. 2020;17(9):e1003346. doi:10.1371/journal.pmed.1003346.
