# Supplementary material for: Test-trace-isolate-quarantine (TTIQ) intervention strategies after symptomatic COVID-19 case identification
Source: PLoS One. 2022 Feb 11;17(2):e0263597. doi: 10.1371/journal.pone.0263597 (PMC8836351; doi:10.1371/journal.pone.0263597)
Supplement: S2 Appendix — Impact of asymptomatics. (PDF) [file pone.0263597.s002.pdf]

S2 Appendix. Supplementary results – Impact of asymptomatics

For manuscript "Test-trace-isolate-quarantine (TTIQ) intervention strategies after symptomatic COVID-19 case identification"

Peter Ashcroft[1*], Sonja Lehtinen[1], Sebastian Bonhoeffer[1*]

1 Institute of Integrative Biology, ETH Zurich, Switzerland

* peter.ashcroft@env.ethz.ch (PA); seb@env.ethz.ch (SB)

Impact of asymptomatics

The relative contribution to transmission of asymptomatics versus symptomatics is captured by the parameter α, which we define as the fraction of transmission attributable to asymptomatic individuals in the absence of TTIQ [Eq (S1.2) in S1 Appendix]. This fraction is difficult to estimate empirically. However, it has been observed that approximately 20% of infections are asymptomatic, and that asymptomatically-infected individuals have a lower risk of onward transmission [1]. Hence we expect α to lie somewhere in the region $0\% \leq \alpha \leq 20\%$, but with substantial uncertainty in this estimate.

By varying α in our model, we can observe how TTIQ effectiveness depends on the amount of asymptomatic transmission. We repeat the analysis shown in Fig 4 in the manuscript for $\alpha \in \{10\%, 20\%, 30\%, 40\%\}$ to see how the contribution of asymptomatics affects our predictions of TTIQ efficacy (Fig I). We observe the same trends as in Fig 4 in the manuscript across the different ranges of α: increasing f and decreasing Δ_1 are the most effective strategies to reduce R_{TTIQ} below one.

In Fig II, we show that idealised TTIQ (and also just testing & isolation alone) is maximally effective when $\alpha = 0$ (i.e. no transmission from asymptomatic individuals). The reason for this is that identifying index cases underlies all TTIQ processes, and identification is only possible if individuals are symptomatic.

Even for imperfect TTIQ interventions with inaccuracies and delays, the fraction of transmission attributable to asymptomatics plays an important role in the effectiveness of TTIQ. Under testing & isolation alone, the effective reproductive number R_{TI} increases linearly with α, while with additional contact tracing & quarantine the increase is super-linear (Fig. IIIA).

Finally, we note that TTIQ leads to an increase in the fraction of transmissions that are attributable to asymptomatics, when compared to this fraction in the absence of TTIQ (Fig. IIIB). This is because the transmission due to symptomatics is lowered by testing & isolation, but transmission due to asymptomatics is untouched. Furthermore, additional contact tracing & quarantine does not affect this fraction as it prevents transmission equally from asymptomatic and symptomatic individuals, hence the lines in Fig. IIIB are overlapping.

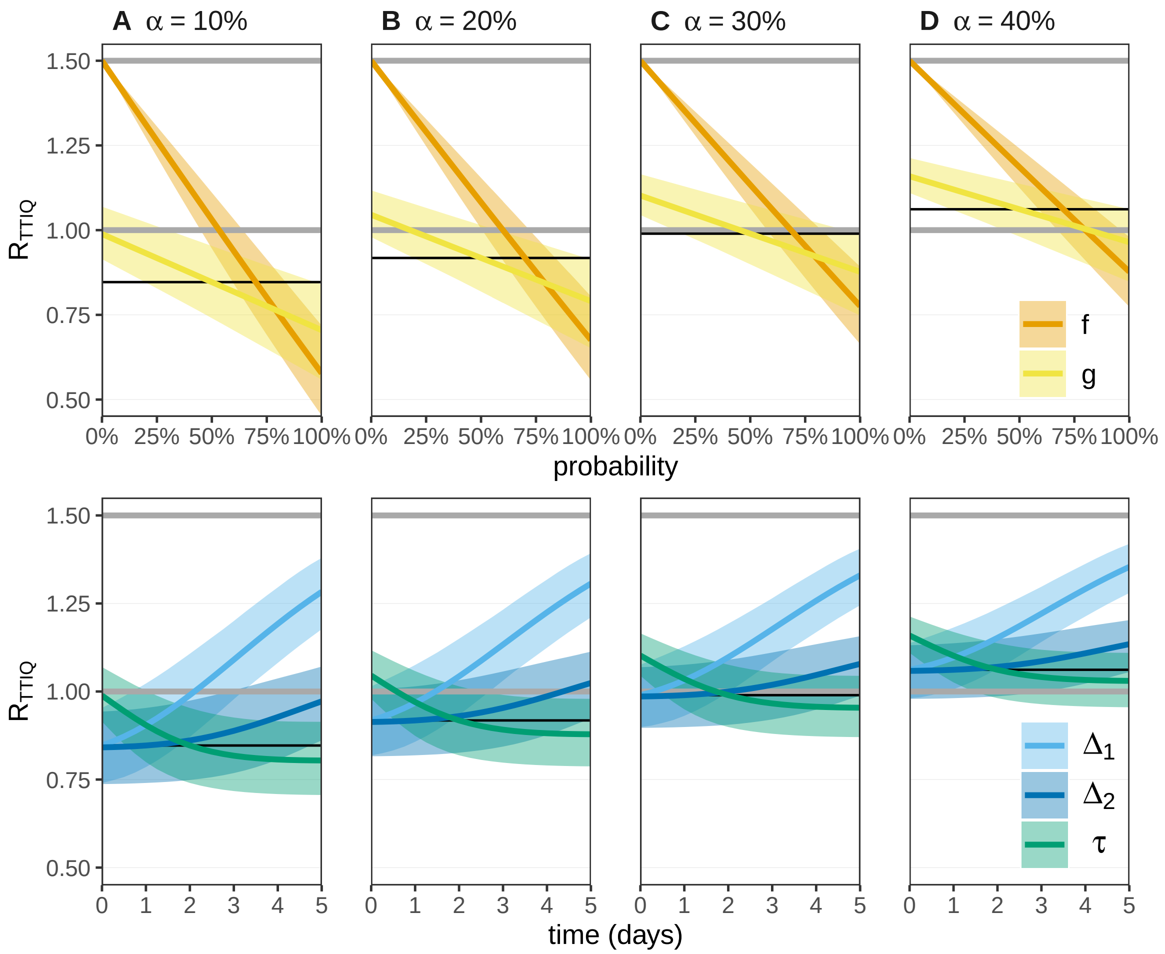

Fig I. The response of the reproductive number R_{TTIQ} to single TTIQ parameter perturbations while varying the fraction of transmission that is attributed to asymptomatic infections α. We set the baseline $R = 1.5$ throughout, which is the intensity of the epidemic in the absence of any TTIQ intervention. We consider perturbations from the focal TTIQ parameter combinations, with $f = 70\%$, $\Delta_1 = 0$ days, $g = 50\%$, $\Delta_2 = 1$ day, and $\tau = 2$ days. R_{TTIQ} for the focal parameter sets are shown as thin black lines. With $f = 0$ (no TTIQ) we expect $R_{\text{TTIQ}} = R$ (upper grey line). We then vary each TTIQ parameter individually, keeping the remaining four parameters fixed at the focal values. The upper panel shows the probability parameters f and g, while the lower panel shows the parameters which carry units of time (days). The critical threshold for controlling an epidemic is $R_{\text{TTIQ}} = 1$ (lower grey line). Asymptomatic individuals are not tested or isolated, but are subject to quarantine after contact tracing. Data provided in S1 Dataset.

References

1. Buitrago-Garcia D, Egli-Gany D, Counotte MJ, Hossmann S, Imeri H, Ipekci AM, et al. Occurrence and Transmission Potential of Asymptomatic and Presymptomatic SARS-CoV-2 Infections: A Living Systematic Review and Meta-Analysis. PLOS Medicine. 2020;17(9):e1003346. doi:10.1371/journal.pmed.1003346.

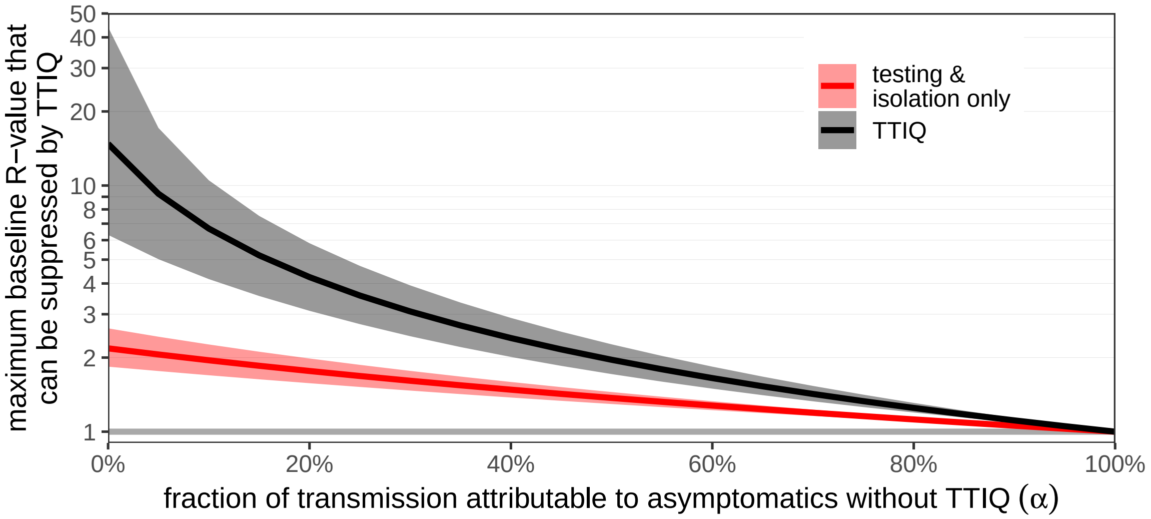

Fig II. The maximum baseline R-value that can be suppressed by TTIQ interventions, as a function of the fraction of transmission that is attributable to asymptomatics α. As $\alpha \to 100\%$, no infecteds develop symptoms and hence no cases are isolated and no contact tracing occurs. In this case, TTIQ has no effect and epidemics are only suppressed if the baseline R-value is already below one. To achieve the maximum level of suppression, each symptomatic individual ($f = 100\%$) would have to isolate immediately at symptom onset ($\Delta_1 = 0$ days), which represents the upper limit of testing & isolation performance. With additional contact tracing, we assume that $g = 100\%$ of contacts of the symptomatic cases who were infected up to $\tau = 5$ days before symptom onset are quarantined immediately ($\Delta_2 = 0$ days). Shaded regions are 95% confidence intervals, representing the uncertainty in the inferred generation time distribution and infectivity profile. Data provided in S1 Dataset.

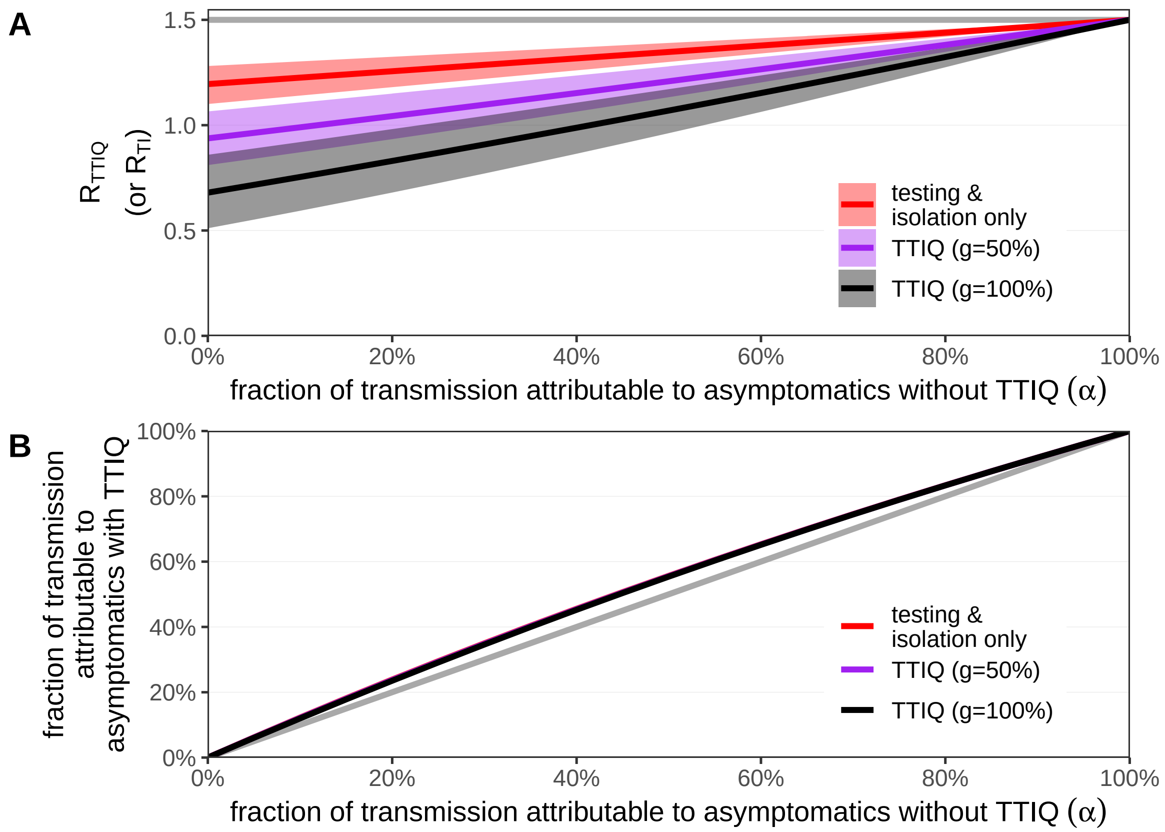

Fig III. Impacts of asymptomatic transmission. A: The impact of the level of asymptomatic transmission on the reproductive number R_{TTIQ}. Here we consider imperfect TTIQ interventions, with $f = 70\%$, $\Delta_1 = 2$ days, $\Delta_2 = 1$ day, $\tau = 2$ days, and a baseline reproductive number of $R = 1.5$. These parameters are equivalent to those used in Fig 4C in the manuscript, along with $g = 50\%$. Here we also consider $g = 0\%$ (testing & isolation only) and $g = 100\%$ (all traced contacts are quarantined). Shaded regions are 95% confidence intervals, representing the uncertainty in the inferred generation time distribution and infectivity profile. B: The fraction of R_{TTIQ} from panel A that is attributable to asymptomatic infection, as described by Eq (S1.15) in S1 Appendix. The diagonal grey line represents the fraction of transmission attributable to asymptomatics without TTIQ interventions. Hence, the TTIQ intervention increases the fraction of transmission that is attributable to asymptomatics. The lines for testing & isolation only, $g = 50\%$, and $g = 100\%$ are overlapping. Data provided in S1 Dataset.
