# Supplementary material for: Test-trace-isolate-quarantine (TTIQ) intervention strategies after symptomatic COVID-19 case identification
Source: PLoS One. 2022 Feb 11;17(2):e0263597. doi: 10.1371/journal.pone.0263597 (PMC8836351; doi:10.1371/journal.pone.0263597)
Supplement: S3 Appendix — Overdispersion. (PDF) [file pone.0263597.s003.pdf]

S3 Appendix. Supplementary results – Overdispersion

For manuscript “Test-trace-isolate-quarantine (TTIQ) intervention strategies after symptomatic COVID-19 case identification”

Peter Ashcroft[1*], Sonja Lehtinen[1], Sebastian Bonhoeffer[1*]

1 Institute of Integrative Biology, ETH Zurich, Switzerland

* peter.ashcroft@env.ethz.ch (PA); seb@env.ethz.ch (SB)

Overdispersion

We have focused our analysis around a fixed baseline reproductive value R; i.e. all individuals would infect R contacts in the absence of TTIQ. Now let us assume that the reproductive value per individual is sampled from an overdispersed negative binomial distribution with dispersion parameter k and mean R. In particular, let the index case infect R_I secondary cases in the absence of TTIQ, where

$$R_I \sim \mathrm{NB}(k, k/(k+R)). \tag{S3.1}$$

Now each secondary case $i \in \{1, 2, \ldots, R_I\}$ infects $R_{S,i} \sim \mathrm{NB}(k, k/(k+R))$ tertiary cases such that, in the absence of TTIQ,

$$n_3 = \sum_{i=1}^{R_I} R_{S,i} \sim \mathrm{NB}(kR_I, k/(k+R)), \tag{S3.2}$$

This distribution follows from the fact that negative-binomially distributed numbers can be represented as the sum of k geometrically distributed numbers, and so the sum of negative binomials is also a negative binomial with appropriate size parameter.

The impact of overdisperal of the contact number distribution in the model is only felt on the baseline reproduction number R, which is factored-out of the expressions for the number of secondary or tertiary cases in the presence of TTIQ:

$$n_2 = R_I \times F(f, \Delta 1), \tag{S3.3}$$

$$n_3 = \left(\sum_{i=1}^{R_I} R_{S,i} \right) \times G(f, \Delta_1, g, \Delta_2, \tau). \tag{S3.4}$$

Therefore, the variance of n_2 will be directly proportional to the variance of R_I, while the mean will be unchanged from the fixed-R approach that we have used.

Our reproductive number, which is defined as the ratio $R_{\text{TTIQ}} = n_3/n_2$, then follows the distribution

$$\frac{n_3}{n_2} = \frac{X(R_I)}{R_I} \times \frac{G(f, \Delta_1, g, \Delta_2, \tau)}{F(f, \Delta 1)}, \text{ where } \begin{cases} X(R_I) \sim \text{NB}(kR_I, k/(k+R)) \\ R_I \sim \text{NB}(k, k/(k+R)). \end{cases} \tag{S3.5}$$

In Fig. I, we see that the expectation value of n_3/n_2 (which is $\text{E}[X(R_I)/R_I]$ in the absence of TTIQ and scaled by a constant independent of k otherwise) is equal to R, while the variance of n_3/n_2 has the same k-dependent shape as the negative binomial (n_2), but with a slightly lower magnitude. Hence, overdispersal does not affect our mean predictions, but it could lead to increased variance/uncertainty in those predictions.

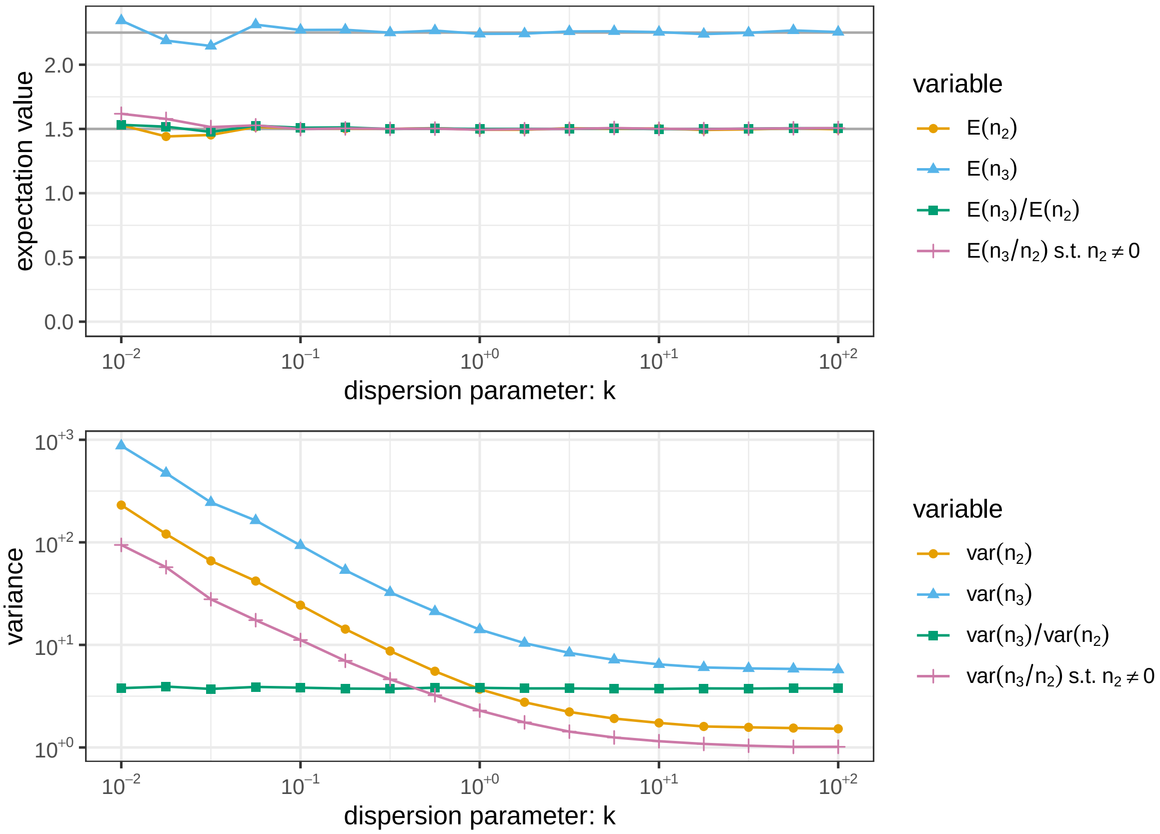

Fig I. The impact of overdispersion on the effective reproduction number. The number of contacts per index and secondary cases follow the same negative binomial distribution with mean $R = 1.5$ and dispersion parameter k (x-axis). Here we have assumed no TTIQ, such that $F(f, \Delta 1) = G(f, \Delta_1, g, \Delta_2, \tau) = 1$. Data provided in S1 Dataset.
