# Supplementary figures and images for: Test-trace-isolate-quarantine (TTIQ) intervention strategies after symptomatic COVID-19 case identification
Source: PLoS One. 2022 Feb 11;17(2):e0263597. doi: 10.1371/journal.pone.0263597 (PMC8836351; doi:10.1371/journal.pone.0263597)

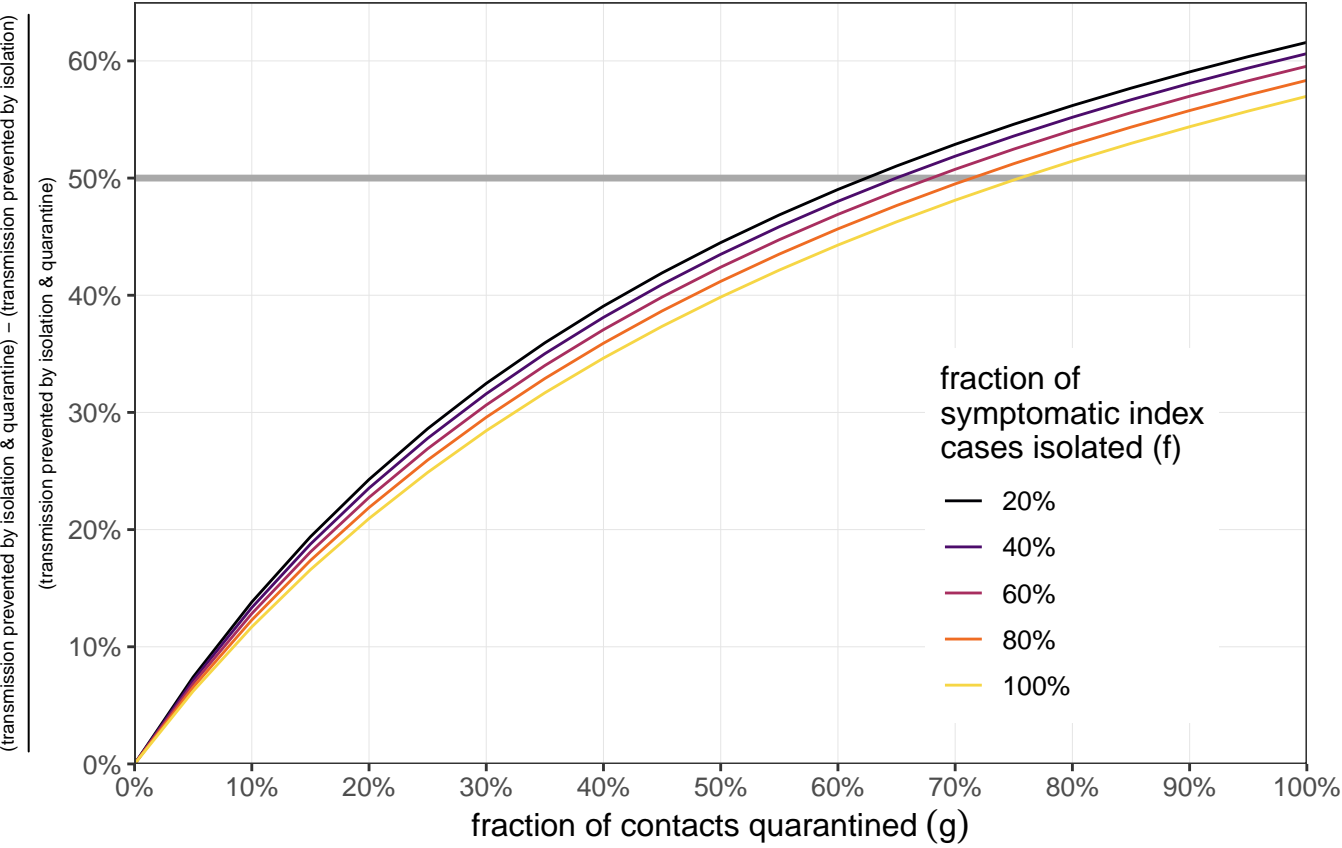

Supplement: S1 Fig — The fraction of prevented transmission that can be attributed to quarantine, rather than isolation. Let RTTIQ(g) be the reproductive number in the presence of TTIQ interventions in which a fraction g of contacts of identified index cases are quarantined. In the absence of TTIQ measures, we expect a reproductive number of R. We then define Y(g) = R − RTTIQ(g) as reduction of transmission due to TTIQ, and Y(0) = R − RTTIQ(0) as the reduction of transmission due only to isolation (i.e. no contact tracing & quarantine). We then define the fraction of prevented transmission due to quarantine as [Y(g) − Y(0)]/Y(g), which we plot as a function of g. Note that we are computing how much extra transmission is prevented by quarantine, which may just be one days worth of transmission before the contact becomes symptomatic and would anyway be isolated. We vary the fraction of symptomatic index cases that are isolated f (colour), and we fix Δ1 = Δ2 = τ = 2 days. We further fix the fraction of transmission that is attributed to asymptomatic infections to α = 20% and R = 1.5 (although the fraction shown is independent of R). Above the horizontal line, more transmission is prevented by quarantine than by isolation. Data provided in S1 Dataset. (PDF) [file pone.0263597.s004.pdf]

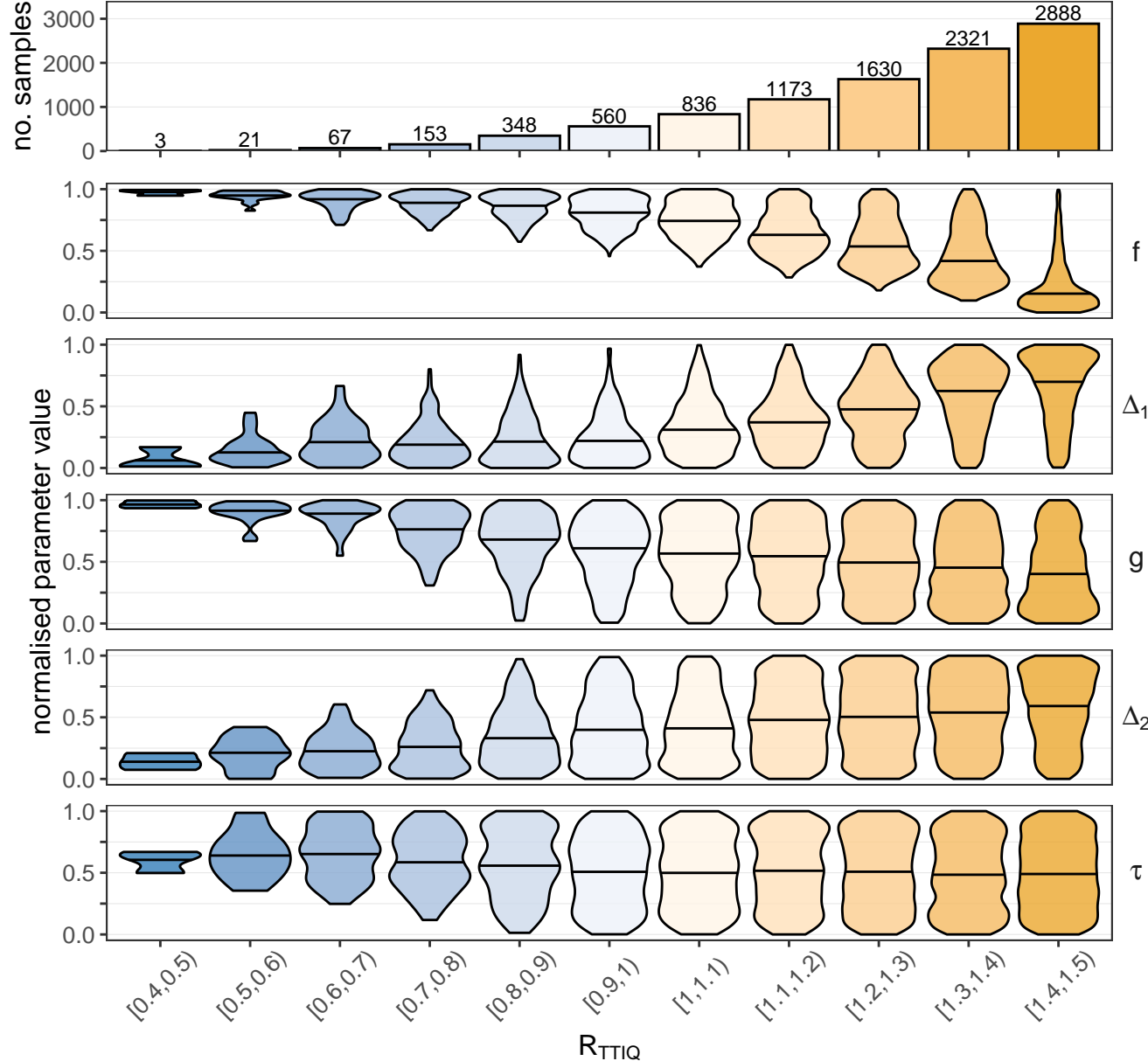

Supplement: S2 Fig — The distributions of (normalised) parameters per categorised group of RTTIQ as used in the LDA analysis in Fig 5 in the manuscript. We uniformly sample 10,000 parameter combinations from f ∈ [0%, 100%], g ∈ [0%, 100%], Δ1 ∈ [0, 5] days, Δ2 ∈ [0, 5] days, and τ ∈ [0, 5] days. The reproductive number RTTIQ is calculated for each parameter combination and categorised into bins of width 0.1 (colour). The upper row shows how many parameter combinations resulted in each category of RTTIQ. The next five rows show how the parameters are distributed within each category, while the horizontal bar shows the median parameter value. We fix R = 1.5 and α = 20%. Data provided in S1 Dataset. (PDF) [file pone.0263597.s005.pdf]

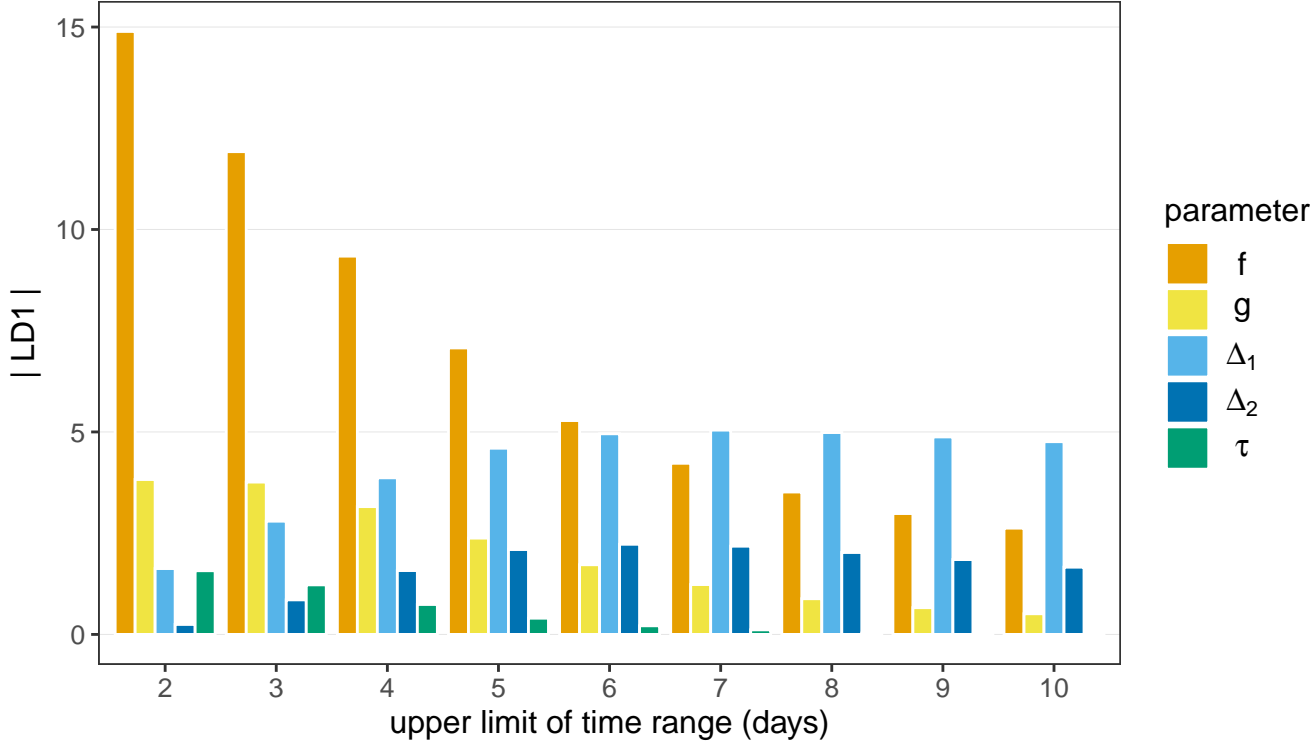

Supplement: S3 Fig — Impact of varying the range from which we sample time-dependent parameters on the LDA output (without quadratic terms). Each bar represents the magnitude of the components of the primary linear discriminant vector (LD1) for each parameter (colour). Data provided in S1 Dataset. (PDF) [file pone.0263597.s006.pdf]

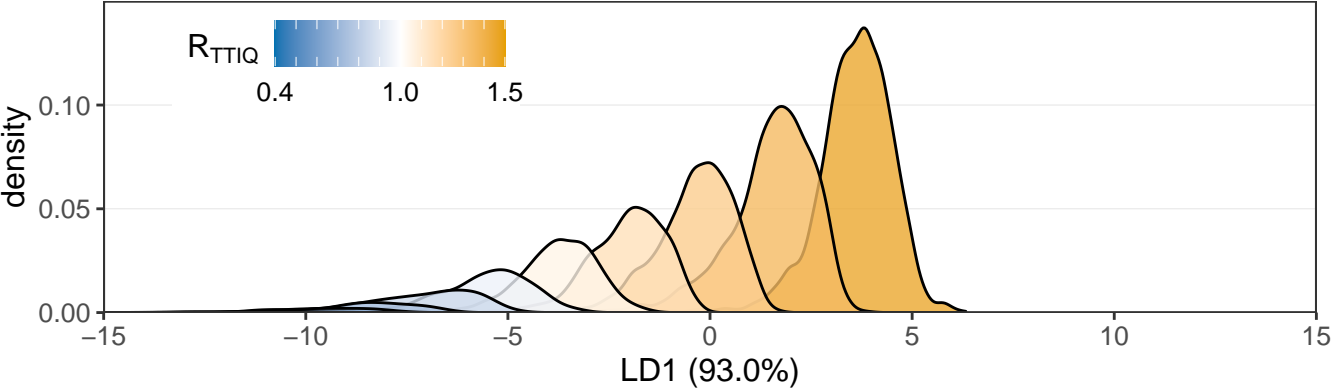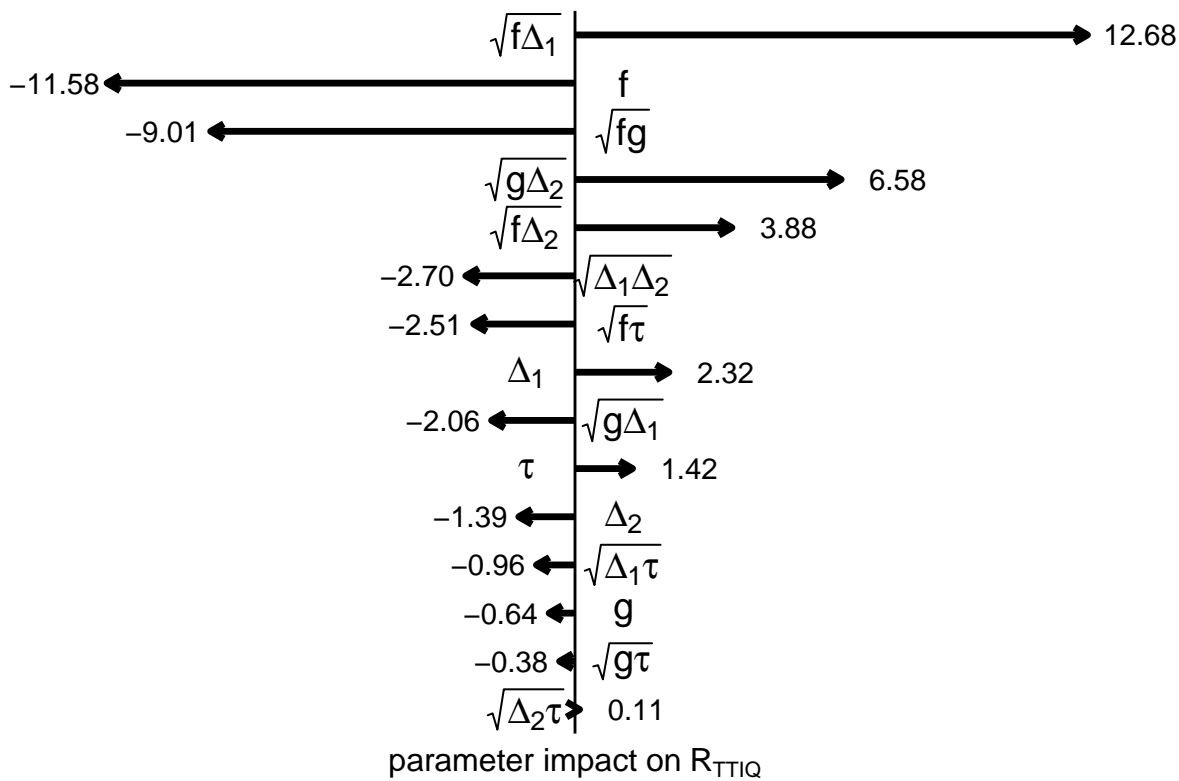

Supplement: S4 Fig — Linear discriminant analysis (LDA) of the impact of TTIQ strategies on the reproductive number RTTIQ, now including quadratic terms. We use the same uniformly-sampled data as in Fig 5 in the manuscript, but now we include the quadratic parameter terms (e.g. f × g) as discriminators too. We take the square root of these quadratic terms to ensure the parameter distributions are not overly skewed. We then use LDA to construct a linear combination (LD1) of the now 15 TTIQ parameters which maximally separates the output categories. We then predict the LD1 values for each parameter combination, and construct a histogram of these values for each category. The lower panel shows the components of the primary linear discriminant vector (LD1). By multiplying the (normalised) TTIQ parameters by the corresponding vector component, we arrive at the LD1 prediction which corresponds to the predicted reproductive number under that TTIQ strategy. Longer arrows (larger magnitude components) correspond to a parameter having a larger effect on the reproductive number. Data provided in S1 Dataset. (PDF) [file pone.0263597.s007.pdf]
