# Supplementary material for: Test-trace-isolate-quarantine (TTIQ) intervention strategies after symptomatic COVID-19 case identification
Source: PLoS One. 2022 Feb 11;17(2):e0263597. doi: 10.1371/journal.pone.0263597 (PMC8836351; doi:10.1371/journal.pone.0263597)
Supplement: Attachment — Submitted filename: response-PLoSONE.pdf [file pone.0263597.s009.pdf]

Response to Reviewer comments:

Test-trace-isolate-quarantine (TTIQ) intervention strategies after symptomatic COVID-19 case identification

Peter Ashcroft[1], **Sonja Lehtinen**[1], **and Sebastian Bonhoeffer**[1]
[1]*Institute of Integrative Biology, ETH Zurich, Switzerland*

Dear Editor,

we here include a description of the changes that have been made to the manuscript after review at PLOS ONE (PONE-D-21-26262). We believe the quality of the manuscript has been improved based on the comments of the two anonymous reviewers, to whom we show our gratitude. In addition to the changes described below, we have modified the structure of the supplementary information (and references thereto) to reflect the house style of PLOS ONE. We hope this revised manuscript is now suitable for publication in PLOS ONE.

Kind regards,

Peter Ashcroft, Sonja Lehtinen, and Sebastian Bonhoeffer

Comments from the Editor

I found this manuscript to be thoughtfully and clearly written. I think the definition of R = R_3/R_2 is a novel and useful metric for evaluating TTIQ. I also like the interactive graphic. Meanwhile, please address all comments made by the reviewers. Reviewer 1's insightful comments should help to enrich the paper. However per PLOS One's publication criteria it is not necessary to pursue additional analyses - provided the model is clearly articulated and limitations are identified. In addition, I like Reviewer 2's suggestion to make code available and to include a paragraph that relates the methodological results to specific public health interventions. Please note that Reviewer 2's listed references on transmission heterogeneity do not need to be included.

Response: We thank the PLOS ONE Academic Editor Dr. Seth Blumberg for considering and carefully reviewing this article, and for their positive comments. As described below, all data and code are publicly available and we included a discussion paragraph to reinforce the public health impact of this work. We have also made clarifications to the model description and the limitations of our branching process approach.

Comments from Reviewer #1:

Summary: In their manuscript, Ashcroft and coauthors propose a branching process model to assess the effectiveness of test-trace-isolate-quarantine TTIQ interventions on the containment of COVID-19. Their findings are overall consistent with the large body of evidence showing that TTI may help curb the spread of infectious diseases — if done properly [1–6]. However, while the authors incorporate great detail into the transmission of COVID-19 by using empirical distributions for

the generation/serial intervals and the time from contagion to symptoms onset, imperfections of the TTIQ interventions and all connection with its real-life implementation and its challenges are overseen (e.g., imperfect isolation, limited contact tracing capacity, the cost-effectiveness of quarantining large fractions of the population [2–4,6]). Below find some observations for improvement.

Response: We thank this Reviewer for carefully and thoroughly reading our manuscript, and for their critical comments which have helped to enrich the paper. We agree that we have focussed on idealised scenarios of TTIQ implementation – this is intentional as we wanted to quantify the upper bound of TTIQ effectiveness provided that the resources are provided and the policies are adhered too. Actually getting people to adhere to isolation/quarantine or having enough contact tracing capacity is a question for sociologists and economists, rather than infectious disease experts. We therefore focus on using the quantified dynamics of transmission, rather than the studies of human behaviour which greatly vary from country to country. We have addressed all detailed queries as described below, and we hope the Reviewer finds our clarifications suitable.

Comment 1.1: Currently, there is no category for recovered/vaccinated individuals — how does epidemic spread affect the baseline reproduction number? How do the authors compute current COVID-19 incidence?

Response: In our model there is no immune class for the individuals. This is compatible with the branching process approach that we apply, which is relevant during early stages of epidemic outbreak when the susceptible population is much larger (formally the population size is infinite) than the combined infected or immune population. The baseline reproductive number in the model therefore is not changed within the analysis – it is a fixed parameter. Of course, the value of this parameter can be changed to represent changes in the epidemiological scenario – decreasing due to increases population immunity and fluctuating due to seasonality and relaxation/imposition of social distancing policies. Our results are therefore illustrated over a range of baseline reproductive numbers.

Due to the framework and our mathematical definition of the reproductive number R_{TTIQ}, the results related to parameter effect/importance are independent of the chosen baseline reproduction number, so for this part of the analysis the choice of R doesn't matter.

Finally, we do not compute COVID-19 incidence in this analysis.

In response to this comment, as well as further comments below, we have explicitly stated the assumptions that the branching process imposes, and we further discuss these assumptions as limitations in the discussion. Specifically, in the first paragraph of *Materials and Methods* we now say: "The branching process model assumes discrete generations of transmission and an infinite population size, such that the expected number of secondary infections per infected is the same across generations. We therefore do not explicitly include the depletion of susceptibles due to death or acquired immunity during epidemic spread and/or vaccination campaigns. The initial infected individual ...". Furthermore, in paragraph 4 of *Materials and Methods* when we introduce the baseline reproduction number, we clarify that it can be influenced by the population's immunity status: "The R-value will also be proportional to the size of the susceptible pool – which can be depleted due to death or acquired immunity – such that epidemic spread and vaccination campaigns will result in a smaller baseline R-value.". Finally, in *Discussion* we describe the limitations of the branching process approach: "Our analysis is, to some extent, limited by the assumptions which underlie the branching process frame-

work. The infinite population size assumption prevents us from computing the fraction of population that is infected, or from a socioeconomic point of view the fraction of population quarantined at a given time. Furthermore, with the branching process we cannot observe long-term effects caused by depleting susceptibles through quarantine, immunity, or death. However, the branching process approach is valid over short time scales (like the two generations of transmission that we calculate), provided that the susceptible population size is much larger than R^2. The effect of susceptible depletion can also be incorporated by lowering the baseline reproductive value R in the model.".

Comment 1.2: What kind of contact tracing is here considered? If manual, there should be a maximum incidence from which no more contacts could be treated or only primary contacts would be prioritized (e.g., $\tau \to 0$ in the notation used in the manuscript) [3,4]. If digital contact tracing was used, how is the threshold defined? What's the cost of quarantine and the fraction of the population currently isolated? see, e.g., [6]

Response: We do not specify between manual versus digital contact tracing, as we are trying to capture the general act of tracing & quarantining by allowing changeable parameter combinations. For example, for digital contact tracing g would increase with the square of the rate of app uptake. As the Reviewer suggests, reducing the duration τ in which contacts during the pre-symptomatic phase are traced, or the fraction of contacts successfully traced g, impact the number and which specific contacts are traced. Hence, reducing τ or g would reflect limited contact tracing capacity.

The costs of quarantine – as computed in Lunz et al. (i.e. the total quarantine days accumulated in a population) or through the fraction of the population quarantined at a given time – are more socio-economical constructs than epidemioogical and therefore we believe that they are beyond the scope of this infectious disease dynamics paper. This is one reason why we opted to use the simpler branching process approach. Because this approach assumes an infinite populaton size, we cannot compute a fraction of individials in quarantine/isolation at a given time. Furthermore, computing a cost of quarantine as done in Lunz et al. is not possible as we don't track individuals who are falsely quarantined: we only track infecteds.

In response, to this comment, we have added to the Materials and Methods that contact tracing can be digital or manual, and that the contact tracing parameters can be adjusted to reflect these different settings: "Furthermore, contact tracing can also achieved through digital app-based technology (Ferretti et al., 2020). The proposed model applies to both manual and digital contact tracing, but we note that we would expect different parameter combinations for digital versus manual contact tracing, for example reduced delays Δ_2 for digital contact tracing (Ferretti et al., 2020; Kretzschmar et al., 2020)."

Comment 1.3: The definition of *symptoms* is ambiguous; do the authors refer to COVID-specific symptoms, like loss of smell/taste or other less common symptoms? [7] This could affect the value of a and α. How do symptoms-specificity relate to testing criteria in the event of high COVID-19 incidence?

Response: Thank you for the clarification, we are indeed focussing on COVID-specific symptoms, which would lead an individual to undergo testing for the presence of SARS-CoV-2. We have modified our text in Materials and Methods to reflect this: "Individuals who develop symptoms that are indicative of COVID-19 can be tested and subsequently isolated from the population." We define symptomatic

versus asymtomatic infection as used in Buitrago-Garcia *et al.* (2020): asymptomatic cases are those who have no symptoms upon initial reporting and had no symptoms at the end of the follow-up period (i.e. 14 days after infection), such that they would not visit a point of care testing facility. This is where our value of $a \sim 20\%$ comes from, although we perform multiple sensitivity analyses over the contribution of asymptomatics. Symptom specificity and finite testing capacity are not features of our current analyses, so we cannot comment on the role of symptom specificity and testing during high incidence periods.

Comment 1.4: How does the baseline reproduction number relate to the data-driven, testing-dependent observed reproduction number? How does the latter relate to R_0, R_{TTIQ}, and R_{TI}? All reproduction numbers should share the tipping point at $R = 1$, but their absolute value depends on the assumptions of the generation interval.

Response: Our baseline reproduction number R would be higher than the currect observed, testing-dependent R_t, as TTIQ has already impacted this observed value. If we had data on current TTIQ parameters (f,g,Δ_1,Δ_2,τ), as well as the observed reproductive value, we could back-calculate the current baseline reproduction number. As the baseline R does account for the presence of hygiene and social distancing measures, as well as the current epidemiological scenario (immunity, seasonality, etc.), it will be lower than the true R_0 (no intervention, fully susceptible population) of SARS-CoV-2. Hence we have $R_t < R < R_0$. The values R_{TTIQ} and R_{TI} represent specific TTIQ efficacy scenarios which satisfy $R_{\mathrm{TTIQ}} \leq R_{\mathrm{TI}} \leq R$. To improve the clarity of the definition of R, we have included a comparison to the basic reproductive number R_0, as well as the observed effective reproductive number: "As we are interested in quantifying the effects of TTIQ strategies, we introduce the parameter R which represents the effective reproductive number of the virus in the presence of interventions such as mask-wearing, social distancing, school closures etc., but in the absence of isolation and quarantine. We refer to this R parameter as *"the baseline R-value in the absence of TTIQ"*, and we have $R \leq R_0$ due to the presence of the non-TTIQ preventative measures. Furthermore, the baseline reproductive number R should be greater than or equal to the currently observed effective reproductive number, which includes the impact of in-place TTIQ measures."

Comment 1.5: Despite being conceptually different, isolation and quarantine are implemented in the same way in the model (i.e., individuals are removed from the infectious pool and do not contribute further to spread). Isolation/Quarantine is deemed imperfect, as individuals share households, choose not to comply with the instructions, or hide their diagnosis because of economic pressure. The above would lead to identified (suspected) and unidentified new infections, contributing further to the spread.

Response: In our model we do account – to some extent – for imperfect interventions with the parameters f and g. We allow these parameters to take on a range of values because we are not confident in their empirical values, due to the effects of non-adherence etc. Of the fraction of the infecteds that are isolated or quarantined, we assume that all transmission is prevented once removed from the infectious pool, while the remaining fraction (asymptomatics, those who were not tested, those with a false-negative test result, and those not found by contact tracers) remain infectious.

The effects of adherence to quarantine can easily be accounted for in the param-

eter g, the probability for a secondary contact to be effectively quarantined. Lack of adherence to isolation can be accounted for in f, as long as this lack of adherence also means that their contacts are not traced. We now expand on these adherence assumptions in Materials and Methods when each parameter is introduced:

f: "For those individuals who are isolated, we assume that they cannot infect further for the remaining duration of their infectious period. This assumption of perfect adherence to isolation once tested positive will lead to an overestimation of TTIQ effectiveness. Any lack of adherence to isolation could be accounted for in the model by reducing f, as long as this lack of adherence also means that their contacts are not traced."

g: "For those who are quarantined, we assume that they cannot infect further for the remaining duration of their infectious period. This assumption of perfect adherence to quarantined once identified through contact tracing will lead to an overestimation of TTIQ effectiveness. However, any lack of adherence to isolation is easily accounted for in the model by reducing g."

Comment 1.6: What are the testing rates and absolute values behind f and g? For f, the sensitivity of self-administrated tests is considerably lower than point-of-care administrated tests [8], and thus higher rates of false negatives would arise. On the other hand, point-of-care tests are limited, and individuals showing symptoms are told not to go there, thus also favoring underreporting. For a given value of f, how many tests per million per day have to be administrated? And for a given value of g and an average number of close contacts per index case, how many calls have to be performed by the tracing agencies before finding a g% of the newly generated cases? Is it reasonable to assume that all of them would be found exactly Δ_2 days after the report? How does the model deal with individuals that are simultaneously identified as close contact and index case?

Response: Testing coverage f and contact tracing success g are likely to be very different between countries. In our experience, it is difficult to attain such information in Switzerland. For these reasons, we chose to keep f and g as free parameters. As our predicted R_{TTIQ} values are linearly-dependent on f and g, it doesn't matter where we are currently with these values: an absolute change in f or g will lead to the same absolute change in R_{TTIQ} (i.e. dR_{TTIQ}/df and dR_{TTIQ}/dg are constant). Following our parameter summary in Materials and Methods, we now describe the rationale for keeping f and g as free parameters: "Due to the high between-country variability of testing coverage (f), contact tracing success (g), and the respective delays, as well as the lack of publicly-available data on these topics, we keep these values as free parameters in our analyses."

In our model we cannot comment on metrics such as number of tests per day as we do not consider a population model. This metric will depend on the current incidence level, which we do not calculate. Furthermore, we assume that positive self-administered (rapid) tests are backed-up by point-of-care confirmatory tests to initiate contact tracing. Any delay is captured in the parameter Δ_1, and any false-negatives of the self-administered tests are included in the fraction f. We now expand upon this in Materials and Methods: "The fraction isolated f can also be reduced by false-negative results based on potentially less-sensitive self-administered tests, which could prevent infected individuals from seeking confirmatory point-of-care tests."

For g, we cannot comment on the number of calls without assuming a value for the number of daily contacts, which is not part of our model.

The constant delay values Δ_1 and Δ_2 are of course simplifying assumptions, and

we discuss the impact of distributed delays in the response to minor comment 1.8 below.

Comment 1.7: I wonder whether it is correct to compare the efficacy of testing-isolating and tracing-quarantining in absolute terms. As I understand it, contact tracing cannot happen without testing; thus, it is conditional to it.

Response: This is another interesting point: should we report the absolute decrease in the reproductive number for the different strategies, or should we report the relative effect? Ultimately it is important to know if an intervention leads to $R < 1$, so we display results in the absolute. However, we expect that different public health authorities would each prefer a different method, so we therefore provide the tools to calculate both. In our model, as R_{TI} and R_{TTIQ} are proportional to the baseline reproductive number R, calculating the relative effect is a straight forward task.

Comment 1.8: There is something odd with Figure S6; I would have expected the trends to be the other way around (if analyzed conditional one to each other). Currently, it seems that to improve the efficacy of contact tracing, we would have to miss more cases in the testing stage.

Response: This is a very interesting point which we can attribute to semantics. First we note that absolute effectiveness (reduction of the reproductive number, $Y(g) = R_{\mathrm{baseline}} - R_{\mathrm{TTIQ}}(g)$ as described in the figure caption) is an increasing function of both f and g: any increase in either of these parameters increases TTIQ effectiveness. Now, when we compare $R_{\mathrm{TTIQ}}(g)$ and $R_{\mathrm{TTIQ}}(0)$ (just isolation), there is an overlap of individuals who would be removed from the transmission pool by both symptomatic isolation and by quarantine following contact tracing. When we take the difference between these R values, as we do in Figure S6 (now S1 Fig), we are just computing how much extra transmission is prevented by quarantine, which may just be one days worth of transmission before the individual becomes symptomatic and would be isolated anyway. By increasing f, we isolate more symptomatic cases and this outweighs the extra transmission prevented by quarantining them one day earlier than they would be isolated. If we were instead to categorise transmission as prevented by quarantine or isolation depending on which event happens first, we would expect to see a reversal with higher f leading to more transmission attributable to quarantine. But ultimately, we are here interested in the extra benefit that quarantine brings to the overall reduction.

In summary, increasing f decreases the extra benefit of contact tracing because increased isolation decreases the transmission potential of infected contacts which could be prevented by quarantine.

We have addressed this issue by relabelling the figure y-axis label as the fraction "((transmission prevented by isolation & quarantine) - (transmission prevented by isolation)) / (transmission prevented by isolation & quarantine)", as well as modifying the caption: "Note that we are computing how much extra transmission is prevented by quarantine, which may just be one days worth of transmission before the contact becomes symptomatic and would anyway be isolated."

Minor comment 1.1: Abstract and throughout the manuscript: currently, it reads "SARS-CoV-2 pandemic", but it should be "COVID-19 pandemic", as the latter refers to the disease.

Response: Thank you for this clarification. We have made approriate changes

to the Abstract, as well as in the Author Summary and Introduction, to rectify this.

Minor comment 1.2: There are parts in the introduction that would better fit (and are redundant with) the discussions section.

Response: We thank the Reviewer for this suggestion. We have now shortened the discussion of previous work in the introduction, and moved the reasoning for differences between outcomes in these studies to the discussion.

Minor comment 1.3: Lines 126–128: missing reference?

Response: Thank you, we have now added Moore et al. and Sonabend et al. as supporting references for the ongoing use of non-pharmaceutical interventions despite vacination coverage.

- Moore, S., Hill, E. M., Tildesley, M. J., Dyson, L., & Keeling, M. J. (2021). Vaccination and Non-Pharmaceutical Interventions for COVID-19: A Mathematical Modelling Study. *The Lancet Infectious Diseases*, 21(6), 793–802, https://doi.org/10.1016/S1473-3099(21)00143-2.
- Sonabend, R., et al. (2021). Non-Pharmaceutical Interventions, Vaccination, and the SARS-CoV-2 Delta Variant in England: A Mathematical Modelling Study. *The Lancet*, 0(0), https://doi.org/10.1016/S0140-6736(21)02276-5.

Minor comment 1.4: Figures in general: perhaps larger tick labels and larger fonts for figure legends

Response: Thank you for the comment. We have adjusted the font sizes in all figures in the main text and appendices to improve readability of tick labels an legends.

Minor comment 1.5: Discussion: (e.g., lines 418–422) Sensitivity analysis is typically included in the supplementary materials, as in the study of Kretzschmar and coauthors [9].

Response: We thank the Reviewer for pointing this out. We were aware of these sensitivity analyses, but we didn't convey our point very clearly in the discussion. While there is a sensitivity analysis to varying the testing coverage in the Suppl. Mat. of Kretzschmar et al., there is no analysis of varying multiple parameters (e.g. testing coverage and isolation delay) at once. In our analyses in which we do this, we find that there are significant interactions between parameters, such that at long delays the effect of changing coverage doesn't have a large effect, but for shorter delays the sensitivity to testing coverage is increased. We now describe this in the discussion with the following text: "...They [Kretzschmar et al.] further showed that the effective reproductive number was insensitive to varying the testing coverage, although only at a fixed delay of four days between symptom onset and index case isolation. Based on our systematic LDA analysis with quadratic parameters (S4 Fig), we know that there is considerable interaction between testing coverage f and isolation delay Δ_1. Therefore, we expect that sensitivity to testing coverage would appear at shorter delay values, and on average across these parameters we show that increasing f has a greater effect on the reproductive number than decreasing Δ_1."

Minor comment 1.6: A sensitivity analysis for the asymptomatic fraction should

also be performed (as it depends on testing criteria and it's likely to impact the effectiveness of testing policies)

Response: We have performed a new sensitivity analysis for the fraction of transmission that is attributable to asymptomatic infections, α, as shown in new Figure I in S2 Appendix. The actual asymptomatic fraction a is absorbed into this α parameter, such that any change in a represents a change in α. Therefore a sensitivity analysis of a would be redundant. This sensitivity analysis shows, as expected, that increased asymptomatic transmission leads to poorer TTIQ performance. Increasing testing coverage still has the largest impact on R_{TTIQ}. Together, we believe that Figures I, II, and III in S2 Appendix provide a comprehensive overview of the effect that asymptomatics have on our predictions.

Minor comment 1.7: How would random testing be implemented in this framework?

Response: Random testing, for example mass or surge testing, or being tested to obtain a COVID certificate, can be implemented by allowing a probability for asymptomatic or presymptomatic cases to be identified as index cases (still accounting for test sensitivity). Because random testing could find secondary contacts before the index case is identified, we would have to correspondingly reduce the size of the contact pool (R) from which we would try to identify and quarantine exposed individuals. Therefore random testing has the triple benefit of increasing index case identification and isolation, reducing the time that these index cases are infectious and non-isolated, and reducing the number of secondary contacts that have to be found by contact tracing. In the discussion, we now add the following text: "In this scenario it would be possible to identify asymptomatic index cases, as well as identifying eventually-symptomatic cases before symptom onset. Through this increased index case identification and isolation, as well as the reduced time that these index cases are infectious and non-isolated, and also reducing the number of secondary contacts that have to be identified by contact tracing, mass/random testing would therefore increase the overall performance of TTIQ."

Minor comment 1.8: Currently, delays are modeled as a fixed parameter. However, how late an individual receives a positive test result is a random variable likely to be overdispersed (and the same for contact tracing). Can the authors perhaps discuss how this would affect their results?

Response: This is an interesting point, which to answer quantitatively would require multiple integrals to average over the distributions of Δ_1 (for index cases and non-identified secondary cases) and Δ_2. We do not perform this additional analysis, but we would expect that the mean reduction in R_{TTIQ} is unchanged (assuming our fixed values Δ_1 and Δ_2 are the means of the respective distributions), but we would generate additional uncertainty in our estimates. In fact, we already average over the infection-to-quarantine duration of secondary contacts (because of distributed infection times), so there would be just a further broadening of this distribution with unchanged mean. The individual effect of distributed delays Δ_1 and Δ_2 can be seen from Figure 4: an index case with a long delay until test result and isolation would lead to a large increase in R_{TI} or R_{TTIQ}, while individual contacts with a long delay until quarantine would lead to a lesser increase in R_{TTIQ}.

In response, we have clarified in Materials and Methods that we consider a fixed delay and added the followig text after introducing all TTIQ parameters: "In all analyses we focus on fixed TTIQ parameter values for all individuals in the branching process, as opposed to sampling each individual's parameters from a

distribution. This simplifies the visualisation and interpretation of results. We expect that the averaged results when using distributed parameters would closely reflect our fixed-value results, but would lead to increased variance/uncertainty in our estimates. Heterogeneity in the individuals' baseline reproductive number (due to contact number and transmission heterogeneities) is addressed in S3 Appendix."

References

[1] Kerr, C. C., Mistry, D., Stuart, R. M., Rosenfeld, K., Hart, G. R., Núñez, R. C., ... & Klein, D. J. (2021). Controlling COVID-19 via test-trace-quarantine. Nature communications, 12(1), 1-12.

[2] Kretzschmar, M. E., Rozhnova, G., & van Boven, M. (2021). Isolation and contact tracing can tip the scale to containment of COVID-19 in populations with social distancing. Frontiers in Physics, 8, 677.

[3] Contreras, S., Dehning, J., Loidolt, M., Zierenberg, J., Spitzner, F. P., Urrea-Quintero, J. H., ... & Priesemann, V. (2021). The challenges of containing SARS-CoV-2 via test-trace-and-isolate. Nature communications, 12(1), 1-13.

[4] Contreras, S., Dehning, J., Mohr, S. B., Bauer, S., Spitzner, F. P., & Priesemann, V. (2020). Low case numbers enable long-term stable pandemic control without lockdowns. arXiv preprint arXiv:2011.11413.

[5] Gardner, B. J., & Kilpatrick, A. M. (2021). Contact tracing efficiency, transmission heterogeneity, and accelerating COVID-19 epidemics. PLOS Computational Biology, 17(6), e1009122.

[6] Lunz, D., Batt, G., & Ruess, J. (2021). To quarantine, or not to quarantine: A theoretical framework for disease control via contact tracing. Epidemics, 34, 100428.

[7] Nasserie, T., Hittle, M., & Goodman, S. N. (2021). Assessment of the Frequency and Variety of Persistent Symptoms Among Patients With COVID-19: A Systematic Review. JAMA network open, 4(5), e2111417-e2111417.

[8] Lindner, A. K., Nikolai, O., Kausch, F., Wintel, M., Hommes, F., Gertler, M., ... & Denkinger, C. M. (2021). Head-to-head comparison of SARS-CoV-2 antigen-detecting rapid test with self-collected nasal swab versus professional-collected nasopharyngeal swab. European Respiratory Journal, 57(4).

[9] Kretzschmar, Mirjam E., et al. "Impact of delays on effectiveness of contact tracing strategies for COVID-19: a modelling study." The Lancet Public Health 5.8 (2020): e452-e459.

Buitrago-Garcia, D., et al. (2020). "Occurrence and Transmission Potential of Asymptomatic and Presymptomatic SARS-CoV-2 Infections: A Living Systematic Review and Meta-Analysis." PLOS Medicine, 17(9): e1003346

Comments from Reviewer #2

Summary: The authors present a well-written and devised study investigating the effect of test-trace-isolate-quarantine (TTIQ) strategies on SARS-CoV2 transmission. Empirical distributions of the generation time, infectivity profile, and incubation period are incorporated into a branching process model with parameters affecting reductions in the distribution of infectivity through time. Early isolation of index cases is found to be the most effective TTIQ strategy and the authors communicate uncertainty in the results exceptionally well throughout. There are a number of typos and a couple aspects of the methods that are a bit unclear. In addition,

further discussion or incorporation of the effects of individual heterogeneities in R should be incorporated. Finally, making the code accessible in addition to the app would be helpful for transparency/reproducibility and future derivative work.

Response: We thank the Reviewer for their kind comments and their constructive criticisms. We have responded to all comments below and modified the manuscript accordingly.

Comment 2.1: Given the theoretical foundation in branching process theory, it's worth investigating the implications of the variance of R on the results in addition to its mean. Previous theoretical work on superspreading and the influence of the dispersion parameter assuming R is negative binomially distributed have found it's important. Especially given the impact of f (fraction of index cases identified), this could lead to interesting insights. If nothing else, I think it's worth a paragraph in the discussion, as my intuition is that it would affect the variance/confidence intervals of the results and not so much the mean.

[1] Blumberg, S., & Lloyd-Smith, J. O. (2013). Comparing methods for estimating R_0 from the size distribution of subcritical transmission chains. Epidemics, 5(3), 131-145.

[2] Blumberg, S., & Lloyd-Smith, J. O. (2013). Inference of R_0 and transmission heterogeneity from the size distribution of stuttering chains. PLoS computational biology, 9(5), e1002993.

[3] Lloyd-Smith, J. O., Schreiber, S. J., Kopp, P. E., & Getz, W. M. (2005). Superspreading and the effect of individual variation on disease emergence. Nature, 438(7066), 355-359.

Response: We agree with the Reviewer that the overdispersal of contacts per index case is an important epidemiological consideration.

Mathematically, in our model, the impact of overdisperal is only felt on the baseline reproduction number R, which is factored-out of the expressions for the number of secondary or tertiary cases. I.e.

$$n_2 = R \times F(f, \Delta 1), \tag{1}$$

$$n_3 = R^2 \times G(f, \Delta_1, g, \Delta_2, \tau). \tag{2}$$

Therefore, the variance of n_2 will be directly proportional to the variance of R, while the mean will be unchanged from the fixed-R approach that we have used. For tertiary cases the calculation is a little more involved. Let us define R_I as the number of secondary cases per index case in the absence of TTIQ, which we assume to follow a negative binomial distribution with dispersion parameter k and mean R: $R_I \sim \mathrm{NB}(k, k/(k+R))$. Now each secondary case $i \in \{1, 2, \ldots, R_I\}$ infects $R_{S,i} \sim \mathrm{NB}(k, k/(k+R))$ tertiary cases in the absence of TTIQ, such that $n_3 = \sum_{i=1}^{R_I} R_{S,i} \sim \mathrm{NB}(R_I \times k, k/(k+R))$. This last step follows from the fact that negative-binomially distributed numbers can be represented as the sum of k geometrically distributed numbers, and so the sum of negative binomials is also a negative binomial with appropriate size parameter. Our reproductive number, which is defined as the ratio n_3/n_2, then follows the distribution

$$\frac{n_3}{n_2} = \frac{X(R_I)}{R_I} \times \frac{G(f, \Delta_1, g, \Delta_2, \tau)}{F(f, \Delta 1)}, \text{ where } \begin{cases} X(R_I) \sim \mathrm{NB}(R_I \times k, k/(k+R)) \\ R_I \sim \mathrm{NB}(k, k/(k+R)). \end{cases} \tag{3}$$

Below in Fig. 1 we show that the expectation value of n_3/n_2 (which is $\mathrm{E}[X(R_I)/R_I]$ in the absence of TTIQ and scaled by a constant independent of k otherwise) is equal to R, while the variance of n_3/n_2 has the same k-dependent shape as the

negative binomial (n_2), but with a slightly lower value. Therefore, we can confirm the Reviewer's intuition that the mean is unaffected, but overdispersion will lead to increased uncertainty in our predictions.

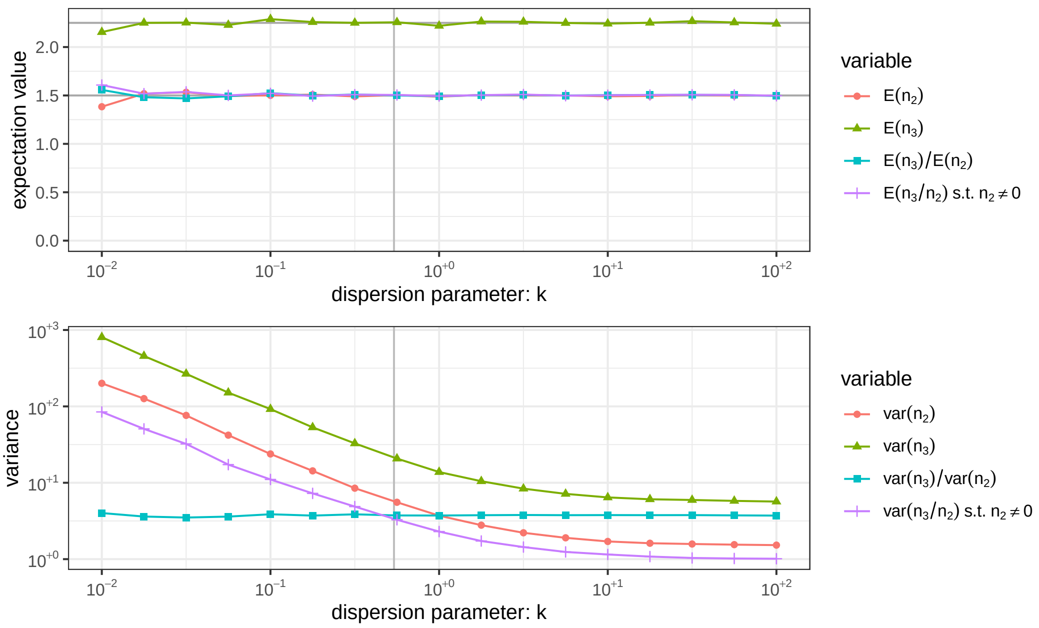

Fig. 1 The impact of overdispersion on the effective reproduction number. The number of contacts per index and secondary cases follow the same negative binomial distribution with mean $R = 1.5$ and dispersion parameter k (x-axis). Here we have assumed no TTIQ, such that $F(f, \Delta 1) = G(f, \Delta_1, g, \Delta_2, \tau) = 1$.

One effect that we do not consider, as also raised by Reviewer 1, is the finite capacity of contact tracing – such that contact tracers can become overwhelmed if an index case is in the tail of the contact number distribution. Such considerations, as decribed in our response to Reviewer 1, are beyond the scope of our paper as we do not want to make assumptions about contact tracing capacity.

In response to this Reviewer's point, we have added the following paragraph to the discussion describing the small limitation imposed by assuming a fixed, rather than overdispersed R-value: "Assuming a fixed value of the baseline reproductive value R is a further limitation of our approach, as the impact of overdispersion of contact number distributions and superspreading is well documented for infectious disease dynamics (Lloyd-Smith et al., 2005). If we were to sample R for the index case and each secondary case from identical overdispersed negative binomial distributions, then the expectation value would be unchanged from our current approach: only the variance/uncertainty in our predictions would increase (S3 Appendix). The equivalence of expectation values could break down if we were to assume a finite capacity of contact tracing, such that the quarantined fraction of contacts of index cases with a large individual reproductive number may be less than g.", as well as S3 Appendix *Supplementary results – Overdispersion* which covers the above reasoning and figure.

Minor comment 2.1: Line 39: Introduce TTIQ acronym first time appearing in summary

Response: Thank you. Corrected.

Minor comment 2.2: Line 64: "were" rather than "are"

Response: Thank you. Corrected.

Minor comment 2.3: Line 79: "Testing and quarantine do not…" rather than "Testing and tracing does not…"

Response: Thank you. Corrected.

Minor comment 2.4: Line 124: Might be worth explaining how this is a methodological advance over Fraser et al (2004) by calling attention to a few specific details

Response: Thank you for the comment. In response to Reviwer 1's comments (Minor 1.2), we have removed this reference from the introduction, and it is only present in the Discussion in the following form: "This difference can be attributed to Ferretti et al. (2020b)'s use of Fraser et al. (2004)'s approach to model contact tracing and isolation as independent events (i.e. tracing an index cases' contacts says nothing about whether the index case has been isolated). Although this assumption leads to analytically tractable predictions of the reproductive number under TTIQ, it also leads to an overestimation of contact tracing's impact (Fraser et al., 2004). Our approach can therefore be considered as a methodological advance over Fraser et al. (2004) and should be employed in the analysis of future epidemic scenarios."

Minor comment 2.5: Line 145: Seems like the definition of the infectivity profile should include reference to symptomatic vs asymptomatic infection and clarify how it is defined in the case of an asymptomatic infection. Looks like it's mentioned in the discussion, but probably worth stating in the methods

Response: The Reviewer raises an important point: the infectivity profile is not defined for asymptomatic cases as there is no symptom onset time to serve as the reference point. However, for our mathematical analysis this does not matter: asymptomatic index cases are not isolated, so we do not have to truncate the infectivity profile to determine how many secondary infections occur. Hence we integrate over the full infectivity profile $p(t|\theta_p)$, which, as a probability density function, has an integral of one. The number of secondary infections per asymptomatic index case is $R_a = \alpha R$. As stated in the discussion, we do make the simplifying assumption that the generation time distribution $q(t|\theta_q)$ is equivalent between symptomatic and asymptomatic infecteds, although the number of secondary cases per infected is different (R_s versus R_a). We have now clarified this in *Materials and methods*: "The fraction of transmission that occurs before symptom onset in symptomatically-infected individuals is defined by the cumulative infectivity profile (or generation time) up to the time of symptom onset. The infectivity profile and incubation periods are undefined (and unnecessary) for asymptomatic cases, and in the model we make the simplifying assumption that the generation time distribution is the same between asymptomatic and symptomatic cases."

Minor comment 2.6: Figure 1 is quite nice, but it's a bit unclear what the y-axis is meant to represent. Is it the probability of generating a new case at time t? Such that infecteds are more likely to generate new cases around the time of their symptom onset? This makes sense, even as it's not explicitly incorporated into the distributions used to generate Fig 1, but it's worth a bit more explicit discussion. Also, in the event that the y axis does represent this transmission probability, it might be worth incorporating this into the timing of secondary cases in the figure such

that more of the secondary cases are generated around the time of highest infectiousness, but this is just a minor suggestion that might not be worth the effort to re-configure the figure.

Response: The y-axis in Figure 1 is indeed the probability density of generating a new case at time t, which when appropriately scaled can also be interpreted as the infectiousness of the infected individual at a specific time. The illustrated distributions are schematic representations of the infectivity profile and/or generation time distributions, which we now describe in the caption: "The distributions shown here are schematic representations of the infectivity profile and/or generation time interval, which are quantitatively displayed in Fig I in S1 Appendix. These distributions reflect an individual's infectiousness as a function of time."

Furthermore, we have now included y-axis labels in Figure 1 such that it is clear that we are plotting the probability of generating a new case at time t. We have also clustered the infection of secondary contacts around the index case's symptom onset time, reflecting the index case's infectivity profile.

Minor comment 2.7: Code and details such as coding language and additional software packages used should be made available in addition to the app

Response: All code used to generate this manuscript, including the manuscript text and code, is publicly available at https://github.com/ashcroftp/COVID-TTIQ and archived at https://doi.org/10.5281/zenodo.4701470 as a single R-markdown document, which is now made clear in the data availability statement. Furthermore, we have stored the data presented for each figure to be generated as human-readable CSV files.

Minor comment 2.8: Figure 2A: consider using color-blind friendly color palette

Response: Thanks for the suggestion, we have switched to the Okabe-Ito palette orange (#E69F00; high reproductive number) and blue (#0072B2; low reproductive number) in figures 2A, 5, S2, and S4.

Minor comment 2.9: Not sure if possible as I'm not familiar with LDA, but would be very interesting to perform the same analysis on pairs of parameters, i.e. could answer: f is most impactful, but what is the most impactful parameter that interacts with f?

Response: This is an interesting idea. As LDA forms a linear map between the output (R_{TTIQ}) and parameter inputs, parameter interactions are not accounted forby definition. To account for parameter interactions, one could include quadratic terms of the form e.g. $f \times g$ as discriminators in the LDA. While this captures interactions between parameters, it makes the results harder to interpret as each individual parameter is present in five terms of the LDA projection. We do include this new analysis in Supplementary Figure S4, and we add the following to the results section following Figure 5: "Furthermore, as a linear approximation the LDA does not capture the effect of covariance between parameters. To capture these parameter interactions, we can also include quadratic terms (e.g. $f \times g$) as independent parameters in the LDA. From this analysis (S4 Fig), we see that the terms $f \times \Delta_1$ and $g \times \Delta_2$ correlate positively with R_{TTIQ}, such that increasing the delays Δ_1 and Δ_2 can negate the increase in TTIQ efficacy that is bought by increasing f or g, respectively."

Minor comment 2.10: Line 394: "than" rather than "that"

Response: Thank you. Corrected.

Minor comment 2.11: Line 452: "contacts" rather than "contracts"

Response: Thank you. Corrected.

Minor comment 2.12: Might be worth adding a paragraph in the discussion suggesting ways to enact the most impactful TTIQ interventions. This would help translate the results into actionable policy for public health practitioners that may not fully understand the methods and approach. Widely available rapid testing for instance could be suggested as a way to increase f.
[1] Larremore, D. B., Wilder, B., Lester, E., Shehata, S., Burke, J. M., Hay, J. A., ... & Parker, R. (2021). Test sensitivity is secondary to frequency and turnaround time for COVID-19 screening. Science advances, 7(1), eabd5393.

Response: We agree with the Reviewer and Editor that this is a great idea. We have added the following text in the first paragraph of the discussion: "From a public health perspective, increasing the identification and speeding up the isolation of symptomatic index cases could be achieved through widely-available rapid testing. Despite the potentially lower sensitivity of rapid tests compared to RT-PCR tests, their effectiveness at reducing transmission has been demonstrated in simulation studies of index case isolation (Larremore et al., 2021)."
